# Cyanopeptolins and Anabaenopeptins Are the Dominant Cyanopeptides from *Planktothrix* Strains Collected in Canadian Lakes

**DOI:** 10.3390/toxins16020110

**Published:** 2024-02-17

**Authors:** Catrina D. Earnshaw, David R. McMullin

**Affiliations:** Department of Chemistry, Carleton University, Ottawa, ON K1S 5B6, Canada

**Keywords:** cyanobacteria, *Planktothrix*, cyanopeptides, metabolomics, GNPS molecular networking

## Abstract

Common bloom-forming cyanobacteria produce complex strain-specific mixtures of secondary metabolites. The beneficial and toxic properties of these metabolite mixtures have attracted both research and public health interest. The advancement of mass spectrometry-based platforms and metabolomics data processing has accelerated the identification of new metabolites and feature dereplication from microbial sources. The objective of this study was to use metabolomics data processing to decipher the intracellular cyanopeptide diversity of six *Planktothrix* strains collected from Canadian lakes. Data-dependent acquisition experiments were used to collect a non-targeted high-resolution mass spectrometry dataset. Principal component analysis and factor loadings were used to visualize cyanopeptide variation between strains and identified features contributing to the observed variation. GNPS molecular networking was subsequently used to show the diversity of cyanopeptides produced by the *Planktothrix* strains. Each strain produced a unique mixture of cyanopeptides, and a total of 225 cyanopeptides were detected. *Planktothrix* sp. CPCC 735 produced the most (*n* = 68) cyanopeptides, and *P. rubescens* CPCC 732 produced the fewest (*n* = 27). Microcystins and anabaenopeptins were detected from all strains. Cyanopeptolins, microviridins and aeruginosins were detected from five, four and two strains, respectively. Cyanopeptolin (*n* = 80) and anabaenopeptin (*n* = 61) diversity was the greatest, whereas microcystins (*n* = 21) were the least diverse. Interestingly, three of the *P. rubescens* strains had different cyanopeptide profiles, despite being collected from the same lake at the same time. This study highlights the diversity of cyanopeptides produced by *Planktothrix* and further hints at the underestimated cyanopeptide diversity from subpopulations of chemotypic cyanobacteria in freshwater lakes.

## 1. Introduction

Cyanobacteria are an ancient phylum of photosynthetic prokaryotes that occupy freshwater, brackish and marine environments [1]. Through their long evolutionary history, cyanobacteria have developed numerous advantageous traits, including buoyancy regulation, nitrogen fixation, light harvesting, akinete production and secondary metabolite biosynthesis [2,3]. Favorable growth conditions can expedite the aggregation of dense cyanobacteria biomass, leading to “bloom events” [4,5,6]. Anthropogenic-mediated eutrophication and climate change are the major drivers for the global increased intensity and geographic distribution of bloom events [7]. Common bloom-forming genera include *Microcystis*, *Dolichospermum*, *Cylindrospermopsis* and *Planktothrix* [1]. When these blooms senesce, they release mixtures of biologically active secondary metabolites into waterways. Further, the microbial degradation of senescent blooms depletes oxygen, leading to possible hypoxic or anoxic conditions, reducing aquatic ecosystem services [4,7,8].

Cyanobacteria are prolific producers of bioactive secondary metabolites [3,9]. The majority of cyanobacteria metabolites are cyanopeptides generated by NRPS (non-ribosomal peptide synthetase), NRPS-PKS (polyketide synthase) and ribosomally synthesized and post-translationally modified peptide pathways [10,11]. To date, more than 2000 unique cyanobacteria secondary metabolites have been documented [11]. Recombination events within NRPS genes and the presence of tailoring enzymes increase the diversity from these biosynthetic pathways [12,13]. Microcystins are exhaustively studied cyanopeptides because they are potent hepatotoxins and possible human carcinogens (IARC Group 2B) [14]. As such, most jurisdictions monitor for selected microcystins in drinking water and waterways because they negatively impact human, animal and ecosystem health [15]. However, lesser studied, cyanopeptide groups, including aeruginosins, anabaenopeptins, cyanopeptolins, cyanobactins, microginins and microviridins, are co-produced with microcystins [10,16,17]. Many of these cyanopeptide groups are generally known as enzyme inhibitors [9]. Despite this, there is a paucity of data on the environmental concentrations, distributions and modes of action for non-microcystin cyanopeptides [17].

Opportunities to investigate the diversity and distribution of cyanobacteria secondary metabolite exist with the advancement of mass spectrometry platforms and open-source metabolomics data processing tools [18,19]. An increasing number of studies are now considering non-microcystin cyanopeptides when screening the metabolite mixtures released from blooms into freshwater [20,21,22]. Some studies report microcystins and other cyanopeptide groups (i.e., cyanopeptolins and anabaenopeptin) at similar concentrations in freshwater and report their accumulation in animal tissues [23,24,25,26]. Unfortunately, investigations into the cyanopeptide distributions and biological activities are hindered by a lack of reference materials. The present study aimed to gather chemical information for the cyanopeptide profiles of six *Planktothrix* strains collected from Canadian lakes using non-targeted mass spectrometry. This was carried out to broaden our understanding of the cyanopeptide co-production by common bloom-forming species.

## 2. Results

### 2.1. Factor Analysis of LC-HRMS Dataset Shows Strain-Specific Planktothrix Cyanopeptide Profiles

The intracellular cyanopeptide diversity of six filamentous *Planktothrix* strains was investigated using non-targeted mass spectrometry-based metabolomics. Cells from quadruplicate batch cultures of each *Planktothrix* strain were analyzed in ESI-positive mode with a data-dependent acquisition (DDA) experiment. The resulting intracellular extracts generated on days 14 and 28 had similar qualitative cyanopeptide profiles. To simplify cyanopeptide identifications, the factor analysis and GNPS molecular networking exclusively considered samples from day 14. Cyanopeptides were identified by a combination of comparisons of LC-MS (*m*/*z* and RT) with available analytical standards or putatively by the interpretation of MS/MS spectra, published literature and database searching [11]. Product ions used to identify cyanopeptide groups and HRMS data for each identified cyanopeptide are reported in Supplemental Materials.

**Figure 1:**
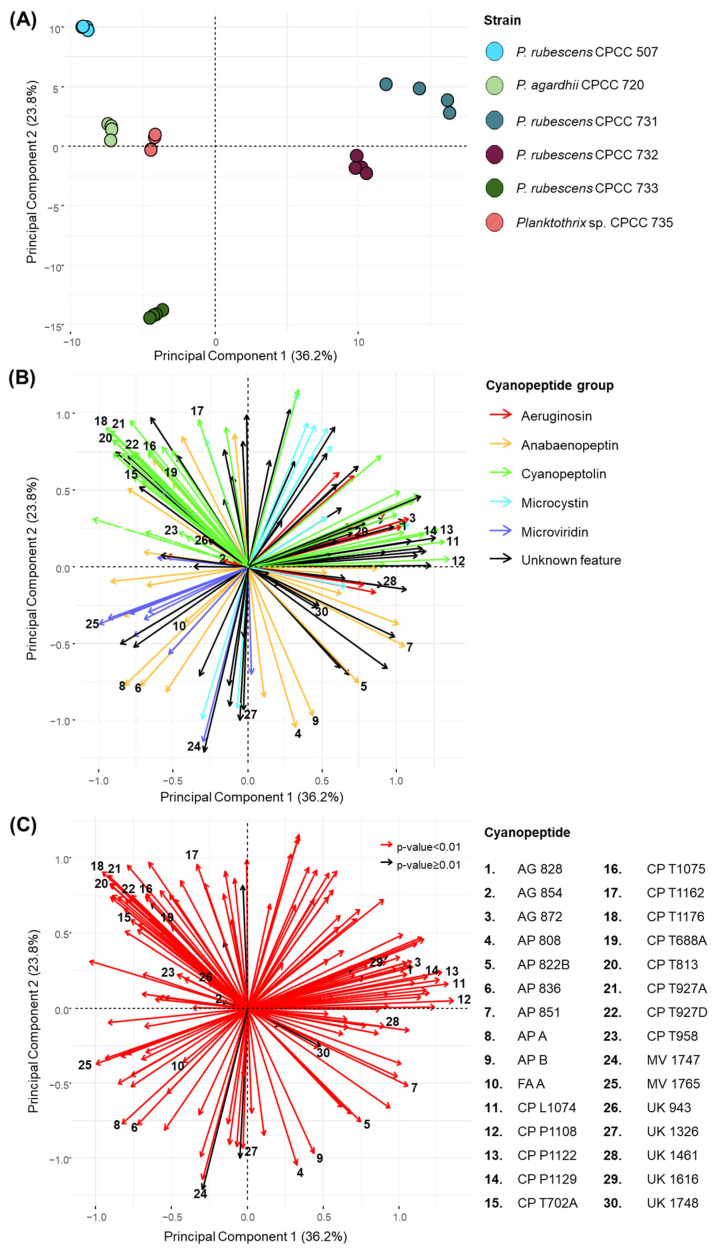
PCA (**A**) plot of intracellular features produced by quadruplicate cultures of the six *Planktothrix* strains. The loading plots show the 154 features considered in the PCA including 84 of the 225 cyanopeptides detected in this study. Cyanopeptide groups are colour coded in the middle loading plot (**B**). For loading plot (**C**), red vectors indicate statistical significance in contribution to variance of the studied *Planktothrix* strains considered in the PCA (*p* < 0.01, Kruskal–Wallis test with Benjamini–Hochberg correction); black vectors have no significant variation (*p* ≥ 0.01). Thirty features are labelled in (**B**,**C**) corresponding to the cyanopeptide legend.

Principal component analysis (PCA) was performed to visualize the cyanopeptide variation between the six studied *Planktothrix* strains (Figure 1). The DDA dataset was deconvoluted by removing features below a 1 × 10^6^ peak intensity threshold, outside 590–1210 *m*/*z*, outside a 2.2–5.6 min retention time window and from the media blank. Features corresponding to redundant ESI adducts and isotopic peaks were also manually removed. The PCA considered 154 features, including 84 of the total 225 cyanopeptides identified: 10 aeruginosins, 20 anabaenopeptins, 31 cyanopeptolins, 12 microcystins and 11 microviridins. Most identified cyanopeptides not included in the factor analysis did not reach the peak threshold. The PCA plot (Figure 1A) showed that each *Planktothrix* strain grouped separately, indicating that each strain generated a unique cyanopeptide profile. Principal component 1 (36.2% variation) mainly describes the variation for *P agardhii* CPCC 720, *P. rubescens* 732 and *Planktothrix* sp. CPCC 735, whereas principal component 2 (23.8% variation) mainly represents the variation for *P. rubescens* CPCC 733 and CPCC 507. The highest internal variance between biological replicates was observed for *P. rubescens* CPCC 731. Factor loading plots (Figure 1B,C) were then constructed to show which cyanopeptide features contribute to the variation observed in the PCA. The coordinate locations of arrows and length dictate the incidence and abundance of cyanopeptides produced by each strain, respectively, and correlate with the PCA. The factor loading (Figure 1B) is color-coded to show how the different cyanopeptide groups influence the PCA. A Kruskal–Wallis test for non-parametric data with a Benjamini–Hochberg correction identified features that statistically contribute to cyanopeptide variation in the PCA plot (*p* < 0.01) (Figure 1C). Thus, 142 of the 154 features considered were statistically relevant (red arrows), meaning most features were drivers of the observed variation in the PCA. The 30 cyanopeptides labelled in the loading plots were determined from volcano plots (Supplementary Materials Figures S3–S8) used to identify the main features differentiating the six *Planktothrix* stains.

Interpretation of HRMS and MS/MS spectra indicated the five strains in the top-left and -right quadrants, or at dimension 1, as *P. rubescens* CPCC 507, *P. agardhii* CPCC 720, *P. rubescens* CPCC 731, *P. rubescens* CPCC 732 and *Planktothrix* sp. CPCC 735, produced cyanopeptolins. *P. rubescens* CPCC 733 is observed separated from all other strains in the bottom-left quadrant of the PCA and did not produce any detectable cyanopeptolins. As expected, green arrows identifying cyanopeptolins are observed in the top-left and -right quadrants of the loading plot (Figure 1B). Further separation of strains in the PCA arises from structural diversity within the cyanopeptolin group. Cyanopeptolin-producing strains in the top-left quadrant, *P. rubescens* CPCC 507, *P. agardhii* CPCC 720 and *Planktothrix* sp. CPCC 735, exclusively produced cyanopeptolins with a threonine-Ahp partial sequence. For example, a green arrow for cyanopeptolin T1176 (feature 18) appears in the top-left quadrant of the loading plot, which is produced by *P. rubescens* CPCC 507 and *P. agardhii* CPCC 720. Alternatively, cyanopeptolin-producing strains in the top-right quadrant of the PCA, *P. rubescens* CPCC 731 and CPCC 732, generate cyanopeptolins that contain Lxx-, phenylalanine- or valine-Ahp moieties. As such, a green arrow for the Lxx-containing cyanopeptolin L1074 (feature 11) appears in the top-right quadrant and is produced by *P. rubescens* CPCC 731 and CPCC 732. While *P. rubescens* CPCC 731 and CPCC 732 have similar cyanopeptide profiles, the major difference is that *P. rubescens* CPCC 731 produces aeruginosins, whereas *P. rubescens* CPCC 732 does not. Aeruginosin production was restricted to *P. rubescens* CPCC 731 and *Planktothrix* sp. CPCC 735. The aeruginosins labelled in the loading plot, aeruginosin 828 (feature 1) in the top-right quadrant and aeruginosin 854 (feature 2) in the top-left quadrant, align with the coordinate location of the respective producing strains, *P. rubescens* CPCC 731 and *Planktothrix* sp. CPCC 735, respectively. *P. rubescens* CPCC 507, *P. agardhii* CPCC 720, *P. rubescens* CPCC 733 and *Planktothrix* sp. CPCC 735 appear in the top-left and bottom-left quadrants of the PCA. Most purple arrows indicative of microviridins are concentrated in the bottom-left quadrant of the loading plot (Figure 1B). Anabaenopeptins and microcystins were produced by all *Planktothrix* strains studied (Table 1).

### 2.2. Molecular Networking with GNPS to Visualize Strain-Specific Cyanopeptide Diversity

**Figure 2:**
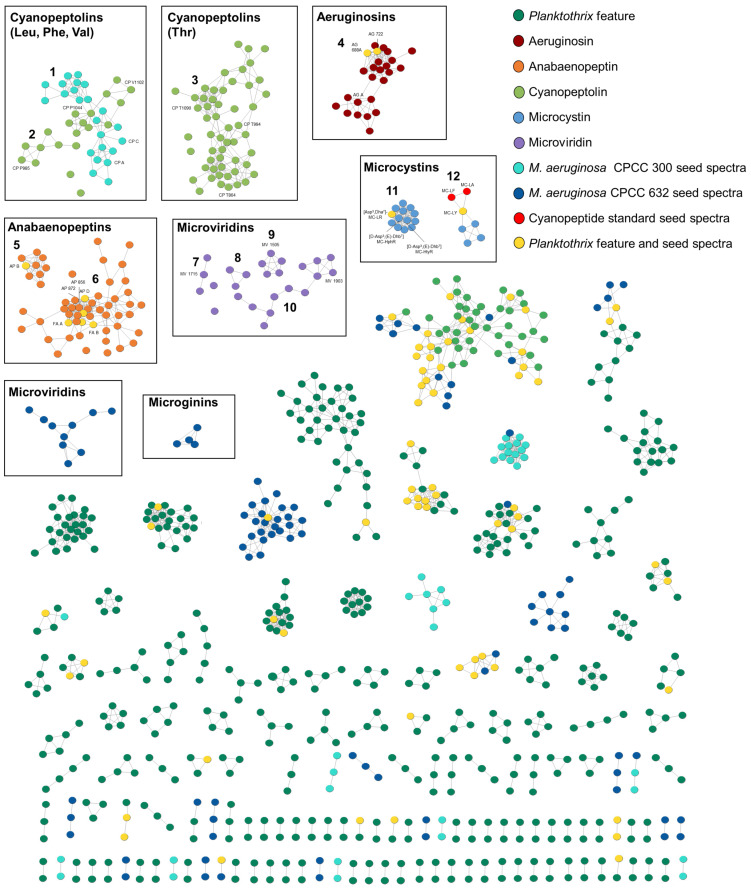
GNPS molecular network generated from feature MS/MS spectra from intracellular *Planktothrix* extracts. Nodes represent a single feature and are colour coded according to the legend. For example, a yellow node was detected in both a *Planktothrix* extract and a seed spectrum. Detailed descriptions of the following cyanopeptide clusters are described in the text: cyanopeptolins (clusters 1–3), aeruginosins (cluster 4), anabaenopeptins (clusters 5–6), microviridins (clusters 7–10), microcystins (clusters 11–12).

GNPS molecular networking was used to visualize and decipher the *Planktothrix* strains’ cyanopeptide profiles (Figure 2). This approach groups features into clusters based on common product ion patterns arising from shared structural features. To target cyanopeptides in the molecular network, similar deconvolution approaches as the factor analysis were employed, except a 1 × 10^5^ peak intensity threshold was used instead of 1 × 10^6^. Cytoscape visualized clusters with a cosine score above 0.7 and 7 matching product ions. To aid in the identification of cyanopeptide clusters in the color-coded molecular network, standards from cyanopeptide groups and two previously studied *Microcystis aeruginosa* cyanopeptide extract seed spectra were added. The resulting molecular network (Figure 2) shows 155 clusters, 923 nodes and 2010 edges. Of the 225 *Planktothrix* cyanopeptides identified, 199 are visualized in the molecular network. Mixtures of cyanopeptolins (clusters 1–3), aeruginosins (cluster 4), anabaenopeptins (cluster 5–6), microviridins (clusters 7–10), microcystins (cluster 11–12) and 9 cyanopeptide single nodes appear in the molecular network. No microginins or prenylagaramides (cyanobactin) were detected, despite their previous characterization from *Planktothrix* strains [27]. Most clusters from the intracellular extracts could not be identified and were not annotated. Identified cyanopeptides not in the molecular network that did not meet the 1E^5^ base peak intensity threshold were identified manually and by diagnostic fragmentation filtering [28]. Seed spectra including standards that appeared as single nodes and not produced by the strains were removed from the network, for example, microcystins -LR and -RR.

#### 2.2.1. Microcystins

All studied strains produced different combinations of microcystins. Microcystin clusters were identified by seed spectra and confirmed by product ions resulting from their characteristic Adda moiety at *m*/*z* 135.0803 and 163.1113 [29]. A total of 21 microcystins were detected from the *Planktothrix* extracts, which were visualized in two clusters (Figure 2; clusters 11–12). Three microcystins were identified manually and are not present in the molecular network, as their intensity was below the base peak threshold. Cluster 11 shows microcystins produced by all strains, excluding *P. rubescens* CPCC 733. This cluster contains (D-Asp^3^, (*E*)-Dhb^7^) MC-HtyR and (D-Asp^3^, (*E*)-Dhb^7^) MC-HphR, which were previously characterized from *P. rubescens* CPCC 507 as well as with [Asp^3^, Dha^7^] MC-LR, which was previously identified from *M. aeruginosa* CPCC 300 [30,31]. Cluster 12 comprises later-eluting, retention time 5.10–5.58 min, microcystins exclusively produced by *P. rubescens* CPCC 733. Three reference materials, MC-LA, -LF and -LY, are visualized in cluster 12; however, only MC-LY was produced by *P. rubescens* CPCC 733. Of the detected cyanopeptide groups, the microcystins represented the least diverse group from the studied strains, highlighting the diversity of understudied non-microcystin cyanopeptides (Table 1).

#### 2.2.2. Cyanopeptolins

The cyanopeptolins were the most diverse cyanopeptide group from the studied strains, and most detected compounds represent putative new structures. Cyanopeptolins are a large group of depsipeptides that all contain an *N*-methyl aromatic residue and a 3-amino-6-hydroxy-2-piperidone (Ahp) moiety. All strains produced unique cyanopeptolin mixtures, with the exception of *P. rubescens* CPCC 733 (Table 1). Further, 74 of the 80 cyanopeptolins detected are distributed between three clusters in the molecular network (Figure 2; clusters 1–3). An additional four cyanopeptolins are visualized as single nodes. Cyanopeptolin clusters were separated in the molecular network based on the identity of residue 3, adjacent to the Ahp moiety. *P. rubescens* CPCC 731 and 732 produced cyanopeptolins that contain leucine/isoleucine (Lxx), phenylalanine or valine at residue 3 (Figure 2; clusters 1–2). Cyanopeptolins with these partial sequences were identified by product ions at *m*/*z* 209.1283 [Lxx–Ahp -H_2_O +H]^+^ and *m*/*z* 181.1331 [Lxx–Ahp -H_2_O -CO +H]^+^, *m*/*z* 243.1121 [Phe–Ahp -H_2_O +H]^+^ and *m*/*z* 215.1174 [Phe–Ahp -H_2_O -CO +H]^+^, *m*/*z* 195.1490 [Val–Ahp -H_2_O +H]^+^ and *m*/*z* 167.1541 [Val–Ahp -H_2_O -CO +H]^+^, respectively [28]. Cluster 1 contains nodes for cyanopeptolins with the Leu–Ahp partial sequence, for example, cyanopeptolins A-C, from *M. aeruginosa* CPCC 300 seed spectra. Alternatively, *P. rubescens* CPCC 507, *P. agardhii* CPCC 720 and *Planktothrix* sp. CPCC 735 produced cyanopeptolins with exclusively threonine at residue 3 (Figure 2; cluster 3). These cyanopeptolins were identified by product ions at *m*/*z* 197.0919 [Thr–Ahp -H_2_O +H]^+^ and *m*/*z* 169.0967 [Thr–Ahp -H_2_O -CO +H]^+^.

#### 2.2.3. Anabaenopeptins

**Figure 3:**
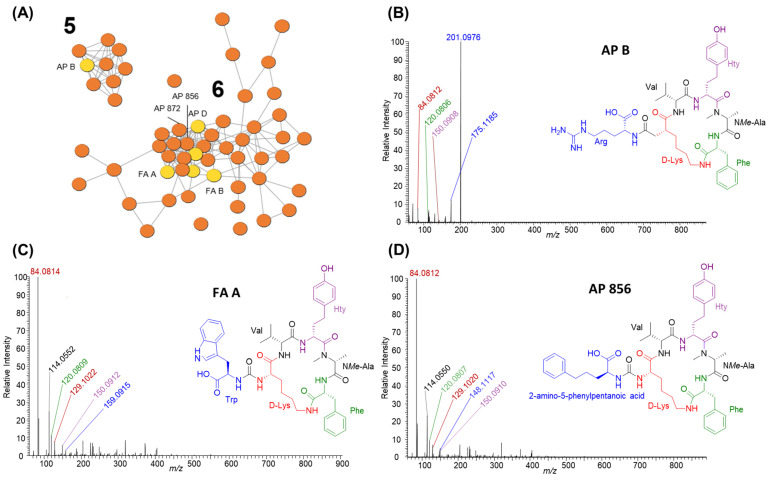
GNPS molecular network clusters 5 and 6 (**A**) showing anabaenopeptin diversity from studied *Planktothrix* (same colour coding as Figure 2). Cluster 5 shows anabaenopeptins with arginine at the exo-position whereas cluster 6 shows anabaenopeptins with several aromatic residues at the exo-position. For example, the MS/MS spectra for anabaenopeptin B (**B**) highlights cluster 5 and ferintoic acid A (**C**) and anabaenopeptin 856 (**D**) highlight cluster 6. Residue immonium and related ions were used to identify putative structures: *m*/*z* 84.0814 (Lys related ion; red), *m*/*z* 159.0908 (Trp; blue), *m*/*z* 175.1192 (Arg + 2H; blue), *m*/*z* 201.0985 (Arg-CO; blue), *m*/*z* 120.0809 (Phe; green), *m*/*z* 148.1117 (2-amino-5-phenylpentanoic acid; blue) and *m*/*z* 150.0910 (Hty; purple).

Anabaenopeptins were the second-most-diverse group of cyanopeptides detected and were produced by all six studied strains. Anabaenopeptins are hexapeptides with a cyclic core, comprising five residues and an exocyclic amino acid connected to the core via a characteristic ureido linkage to D-lysine. Thus, 57 of the total 61 anabaenopeptins detected from studied strains are visualized within two clusters in the molecular network (Figure 2; clusters 5–6). Three additional congeners appear as single nodes. Anabaenopeptins were readily identified by an intense lysine-related ion at *m*/*z* 84.0814 and nodes arising from anabaenopeptins from the *M. aeruginosa* CPCC 632 seed spectrum. The identity of the exo-residue resulted in the two anabaenopeptin clusters in the molecular network (Figure 3). Cluster 5 shows nine anabaenopeptins that contain exclusively an arginine residue at the exo-position, confirmed by product ions at *m*/*z* 201.0985 [Arg -CO +H]^+^ and 175.1192 [Arg + 2H]^+^ (Figure 3B). Cluster 6 shows 47 anabaenopeptins with several aromatic residues, including tryptophan, 2-amino-5-phenylpentanoic acid and 2-amino-5-hydroxyphenylpentanoic acid, at the exo-position. Anabaenopeptins with these exo-residues were confirmed by their immonium ions at *m*/*z* 159.0914, 148.1121 and 164.1070, respectively (Figure 3C,D). We could not confirm anabaenopeptins with phenylalanine, tyrosine, homo-phenylalanine or homo-tyrosine at the exo-position since these residues are regularly incorporated into their cyclic core. The ferintoic acid A (FA A) standard confirmed it was produced by *Planktothrix* sp. CPCC 735, and all strains but *P. rubescens* CPCC 507 produced anabaenopeptin B. Anabaenopeptins 856 and 872 were previously characterized by *P. rubescens* CPCC 507 and are visualized in cluster 6. All strains produced anabaenopeptins with both arginine and aromatic residues at the exo- position, except *P. rubescens* CPCC 507, which only produced congeners with aromatic residues at the exo-position.

#### 2.2.4. Microviridins

Microviridins are N-acyl trideca- (13 residues) and tetradeca- (14 residues) RiPPs. Unlike the other *Planktothrix* cyanopeptide groups investigated, microviridins solely comprise proteinaceous amino acids and are larger structures (1600–1900 Da) [32]. As a consequence, putative microviridins were detected by doubly or triply charged ions in the HRMS dataset and confirmed by the presence of amino acid immonium ions. Dominant immonium ions within microviridin MS/MS spectra are largely attributed to the residues in their variable termini. Thus, 25 of the 35 detected microviridins are visualized in four clusters (Figure 2; clusters 7–10) and as two nodes. Clusters 7–8 show microviridins produced by *P. rubescens* CPCC 507 and CPCC 733, which produced similar congeners. Clusters 9–10 show microviridins from *P. agardhii* CPCC 720 and *Planktothrix* sp. CPCC 735, which also produced similar microviridins. No microviridins were detected from *P. rubescens* CPCC 731 and CPCC 732 extracts. Interestingly, none of the microviridins produced by *M. aeruginosa* CPCC 632 grouped with the *Planktothrix* congeners. None of the detected microviridins aligned with literature and database searches, suggesting they are putative new structures.

#### 2.2.5. Aeruginosins

Aeruginosins are linear tetrapeptides that contain several unusual moieties, including an N-terminal 4-hydroxyphenyllactic acid (Hpla), a 2-carboxy-6-hydroxyoctahydroindole (Choi) and C-terminal arginine derivatives. The tetrapeptide scaffold is commonly sulfated and chlorinated, increasing the group’s structural diversity. Twenty-five aeruginosins are visualized in cluster 4. However, the two aeruginosin-producing strains, *P. rubescens* CPCC 731 and *Planktothrix* sp. CPCC 735, generated discrete assemblages of aeruginosins. All aeruginosins were confirmed by characteristic Choi product ions at *m*/*z* 140.1064 and 122.0962 [33]. Two aeruginosins, 688A and 722, from the seed spectra of *M. aeruginosa* CPCC 632 aided in the identification of the aeruginosin cluster [30]. Three additional aeruginosins were identified by manual interpretation of the data: AG 660A, AG 672 and AG 688B. Aeruginosins shown in cluster 4 primarily contained phenylalanine and tyrosine at position 2, evidenced by product ions at *m*/*z* 284.1268 [Hpla–Phe -CO]^+^ and *m*/*z* 300.1232 [Hpla–Tyr -CO]^+^, respectively. Chlorinated congeners were produced by *Planktothrix* sp. CPCC 735, including AG 722 and four putative new variants (AG 738, AG 756, AG 854 and AG 870). Chlorinated Hpla moieties were confirmed by product ions at *m*/*z* 318.0876 [Cl-Hpla–Phe -CO +H]^+^ and *m*/*z* 334.0838 [Cl-Hpla–Tyr -CO +H]^+^ and examination of isotopic peak profiles. In the case of [Cl-Hpla–Tyr -CO +H]^+^ ions, we could not discriminate if the Cl is on the Tyr or Hpla.

### 2.3. Relative Abundance of Planktothrix Cyanopeptides

**Figure 4:**
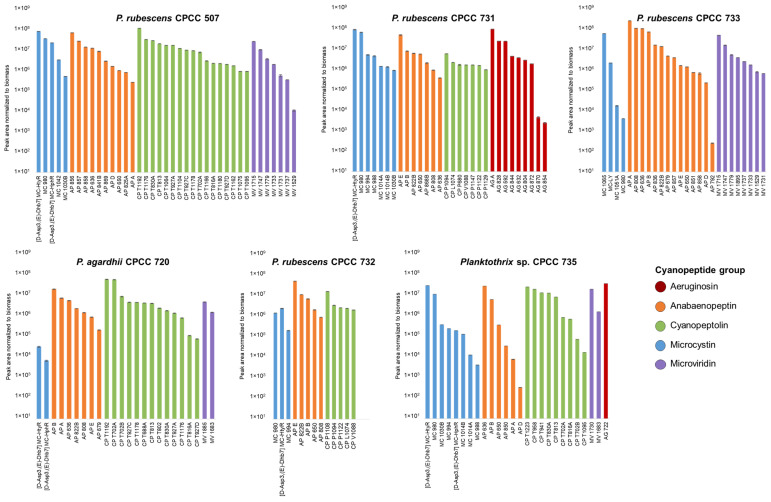
Relative abundances of cyanopeptides from the intracellular extracts of the six studied *Planktothrix* strains. Plot generated using the HRM peak area normalized to dry biomass (peak area/mg) from day 14 extractions. Features are colored based on cyanopeptide group and the average is shown Errors bars show the standard deviation.

The non-targeted metabolomics investigation provided a good understanding of the strain-specific cyanopeptide profiles for the studied *Planktothrix*. There is a lack of cyanopeptide reference materials, especially for non-microcystin groups, required for accurate quantification. To examine the relative amounts of cyanopeptides produced by each strain, HRMS peak areas from quadruplicate cultures were normalized to the cultures’ respective dry biomass (mg). The biomass-normalized peak areas were averaged and plotted to visualize the relative amounts of each cyanopeptide from the intracellular extracts on day 14 (Figure 4). The same deconvolution approaches as the factor analysis were used, resulting in 84 unique cyanopeptides in Figure 4. Following the total cyanopeptides reported in Table 1, *P. rubescens* CPCC 507 had the most cyanopeptides (*n* = 39) included in Figure 4, and CPCC 732 had the fewest (*n* = 13). The normalized relative abundance for most cyanopeptides was similar between strains. While this data representation is not quantitative, it suggests that *Planktothrix* is capable of simultaneously co-producing mixtures of cyanopeptide groups at similar amounts.

## 3. Discussion

*Planktothrix* is a common bloom-forming genus, typically found in temperate freshwater systems. While *Planktothrix* blooms are not as common as *Microcystis* blooms in Canada, both genera are capable of producing strain-specific mixtures of microcystins and other cyanopeptides [1,34]. Historically, most cyanopeptide studies have focused on the microcystins due to their impacts on human and animal health [17]. In this study, we used non-targeted mass spectrometry-based metabolomics to decipher the complex strain-specific cyanopeptide mixtures from the six *Planktothrix* strains (Table 1; Figure 1 and Figure 2; Supplemental Information Table S5). Cyanopeptides detected belong to the microcystin, aeruginosin, anabaenopeptin, cyanopeptolin and microviridin groups. No microginins or prenylagaramides were detected, despite their previous characterization from the genus [27,34]. All strains produced multiple cyanopeptide groups, where microcystins and anabaenopeptins were detected from each strain (Table 1). Cyanopeptolins, microviridins and aeruginosins were detected from five, four and two strains, respectively. Of the 225 total cyanopeptides detected, the cyanopeptolins (*n* = 80) and anabaenopeptins (*n* = 61) represented the most structural diversity. Interestingly, microcystins (*n* = 21) were the least diverse group detected within the studied *Planktothrix* extracts. *Planktothrix* sp. CPCC 735 produced the most cyanopeptides, with 68 unique metabolites detected, and *P. rubescens* CPCC 732 produced the least, 27. Our findings contribute to the growing number of studies showing that *Planktothrix* produces strain-specific cyanopeptide mixtures and highlights the diversity of non-microcystin groups, specifically the anabaenopeptins and cyanopeptolins, from common bloom-forming cyanobacteria [27].

The strain-specific profiles reported from the studied strains are supported by earlier studies of *Planktothrix* cyanopeptides from cultures. Welker et al. 2004 profiled the cyanopeptides from 18 clonal *Planktothrix* isolates from a single water sample collected from a German lake [34]. Most isolates generated distinct cyanopeptide profiles from the groups we detected. Microcystins were only produced by seven of the isolates, and a prenylagaramide was detected from one isolate. Rohrlack et al. 2008 identified four distinct *Planktothrix* chemotypes over a 33-year period from a lake in Norway [35]. These four chemotypes were collected unchanged over the decadal sampling period and could not be explained by fluctuations in light, temperature or macronutrients. They hypothesized that depth regulation or interactions with grazers and pathogens may contribute to the observed chemotypes in the population. Rohrlack et al. 2009 studied 87 *Planktothrix* from 13 Scandinavian lakes, where they detected 46 unique cyanopeptides and 17 chemotypes [36]. Between 1 and 13 cyanopeptides were detected, where cyanopeptolins were the most diverse (*n* = 21), followed by anabaenopeptins (*n* = 6), aeruginosins (*n* = 6), microcystins (*n* = 5), microginins (*n* = 2) and microviridins (*n* = 2). *Planktothrix* chemotypes recurred in specific lakes, suggesting that chemotypes can be present within populations for decades. *P. rubescens* CPCC 731, 732 and 733 were all isolated from Vert Lake, QC, Canada, in October 2008, and each produced different cyanopeptide mixtures. These data support Rohrlack et al. [36], who identified multiple *Planktothrix* chemotype subpopulations collected from the same lakes. More recently, Tiam et al. 2019 used genomics and metabolomics to examine the cyanopeptide profiles of four *Planktothrix* strains [27]. There was concordance between the genomic and metabolomics data, where most cyanopeptides represented putative new structures. Again, unique cyanopeptide mixtures were generated by each strain; however, some strains produced microginins and prenylagaramides. Aeruginosins and cyanopeptolins were universally produced, and both microcystins and anabaenopeptins were detected from three strains. Kurmayer et al. 2015 suggested that cyanopeptide profiles are dependent on speciation processes and that within a lineage, the likelihood of diverse cyanopeptide profiles increases with geographic distance [37]. To the best of our knowledge, none of the reported cyanopeptide profiles from studied *Planktothrix* strains have the same unique cyanopeptide composition (except within chemotypes from the same source).

There have been fewer reports of non-microcystin cyanopeptides from *Planktothrix* blooms. Erratt et al. (2022) investigated a deep cyanobacteria layer within Sunfish Lake in Ontario, Canada [38]. The layer predominantly consisted of *P. isothrix* and *P. rubescens* to a lesser extent. However, *P. rubescens* exhibited a higher cyanotoxin cell quota than *P. isothrix*. Maximum concentrations of detected microcystins and anabaenopeptins were 0.03 μg L^–1^ and 2.5 μg L^–1^, respectively. Interestingly, anabaenopeptins were detected at higher concentrations than microcystins. Detected cyanopeptides included MC-LA, MC-LY, MC-LW, [D-Asp^3^] MC-LR and anabaenopeptins A and B. We detected many of these cyanopeptides from the strains studied here. For example, MC-LY was produced by *P. rubescens* CPCC 733, anabaenopeptin A was produced by *P. rubescens* CPCC 507, *P. agardhii* CPCC 720, *P. rubescens* CPCC 733 and *Planktothrix* sp. CPCC 735, and anabaenopeptin B was produced by all strains but *P. rubescens* CPCC 507. Deep cyanobacteria layers are not commonly reported; thus, lower incident rates may reflect a monitoring bias [38]. *P. rubescens* preferentially occupies deeper water layers in mesotrophic lakes and, therefore, its occurrence may be underestimated [39]. Although microcystin concentrations from the Sunfish Lake cyanobacteria layer were below drinking water thresholds, environmental changes favoring *P. rubescens* may induce increased cyanopeptide water concentrations. Zastepa et al. (2021) characterized nearshore deep chlorophyll layers from Twelve Mile Bay and South Bay, two meso-oligotrophic embayment’s along Eastern Georgian Bay of Lake Huron [40]. Both embayment’s deep chlorophyll layers were predominantly composed of *Planktothrix* cf. *isothrix*. Measurable concentrations of microcystins (max. 0.4 µg/L), anabaenopeptins (max. ~7 µg/L) and cyanopeptolins (max. 1 ng/L) were detected. MC-LA, which was not detected from our strains, was the most frequently detected and at the highest concentrations. McKay et al. 2020 examined a bloom co-dominated by *Aphanizomenon flos-aquae* and *Planktothrix agardhii* in the Thames River, Ontario [41]. Low microcystin concentrations (<1.5 µg/L) were detected in samples from the Thames River mouth by ELISA; however, microcystins were not detected from bloom samples by mass spectrometry-based analysis.

Historically, non-microcystin cyanopeptides have generally been considered “non-toxic” and enzyme inhibitors, mainly serine protease inhibitors [9,17]. The inhibition of serine proteases, digestive enzymes such as trypsin, is hypothesized to modulate interactions with grazers and pathogens. An increasing number of studies are now showing that cyanopeptolins and anabaenopeptins can induce neurotoxic and systemic toxicity in various model organisms [42,43,44,45]. Further, microcystins and anabaenopeptins have been reported at similar concentrations (low µg kg^−1^) in fish muscle tissue [26]. A better understanding of the diversity of metabolites released by blooms into the environment given the expanding literature on their biologically active properties, environmental concentrations and accumulation in animal tissues appears prudent. Future work will include comparisons of cyanopeptide profiles from cultured strains, representing common bloom-forming genera and bloom events in the region. This will guide the cultivation of strains producing common water contaminants to generate cyanopeptide standards for exposure and biological activity assessments.

## 4. Materials and Methods

### 4.1. Planktothrix Strains

Four *Planktothrix rubescens* strains (CPCC 507, CPCC 731, CPCC 732, CPCC 733), *P. agardhii* CPCC 720 and *Planktothrix* sp. CPCC 735 (identity uncertain, previously identified as *Oscillatoria tenuis*) were purchased from the Canadian Phycological Culture Centre (CPCC) at the University of Waterloo, Canada. Collection information and an image for each strain are provided in Supplementary Materials Table S1 and Figure S1. All strains, apart from *P. rubescens* CPCC 507, were maintained in Z8 medium. *P. rubescens* CPCC 507 was grown in a modified WC(ed) medium [46]. Quadruplicate cultures of each strain were grown in 250 mL Erlenmeyer flasks (Pyrex; Stoke on Trent, UK) for either 14 or 28 days. On day 0, 125 mL of sterile media was inoculated with 25 mL of cyanobacteria cells after blending. Cultures were maintained using a light/dark regime of 12 h under cool white, fluorescent light (30 μE m^−2^ s^−1^) in a Conviron E15 growth chamber at 21 °C. Every two days, the flasks were swirled.

### 4.2. Cyanopeptide Extraction

To extract intracellular cyanopeptides, aliquots of *Planktothrix* cells after 14 or 28 days were harvested onto glass microfibre filters (pre-weighed and dried overnight at 40 °C; Whatman, GF/A, diameter 47 mm, ~1.6 µm) with a Millipore filtration apparatus. The glass microfibre filters with the harvested *Planktothrix* cells were dried overnight (40 °C) and were subsequently transferred to glass culture tubes (16 × 100 mm, VWR) with 14 mL of 80% aqueous methanol. Each sample test tube was vortexed and sonicated three times for 30 s intervals. Sample tubes were stored at −80 °C for 40 min and were subsequently allowed to thaw at room temperature. This freeze–thaw cycle, in combination with agitation and sonication, was repeated two additional times to induce cell lysis. Resulting intracellular cyanopeptides in the methanolic extracts were filtered through 0.22 µm PTFE syringe filters (25 mm; ChromSpec, Inc., Brockville, ON, Canada) into amber vials and dried under a gentle stream of nitrogen gas. The dried extracts were reconstituted in 1.5 mL of HPLC-grade methanol and transferred to 2 mL amber HPLC vials (Agilent, Santa Clara, CA, USA), dried under a nitrogen gas and stored at −20 °C until instrumental analysis. Data for the extraction of *Planktothrix* intracellular compounds are reported in Supplementary Materials Table S2.

### 4.3. Acquisition of Non-Targeted LC-(HR)MS/MS Data

Intracellular cyanopeptide extracts were solubilized in 1 mL of 90% aqueous methanol and vortexed for 15 s prior to LC-MS/MS analysis. Non-targeted data were acquired with an Agilent 1290 HPLC (Agilent Technologies, Santa Clara, CA, USA) coupled to a Q-Exactive Orbitrap (Thermo Fisher Scientific, Waltham, MA, USA) mass spectrometer [30]. Extracts were resolved using an Eclipse Plus C18 RRHD column (2.1 × 100 mm, 1.8 µm; Agilent Technologies) maintained at 35 °C and a 10.5 min gradient program optimized for cyanopeptides. Mobile phases (A) and (B) consisted of water with 0.1% formic acid and acetonitrile with 0.1% formic acid, respectively. The method increased from 0% to 35% B over 1.5 min, increased from 35% to 45% B over the next 2.5 min, then increased to 100% B over 2 min, where it was held for 3 min before returning to 0% B over 0.5 min and maintained at 0% B for the final minute. Further, 5 µL of each extract was injected, and a 300 µL min^−1^ flow rate was used.

In positive mode, a heated electrospray ionization source was used with a capillary voltage of 3.9 kV, capillary temperature of 400 °C, sheath gas of 19 units, auxiliary gas of 8 units, S-Lens RF level of 45.00 and a probe heater temperature of 450 °C. (HR)MS data were acquired with a non-targeted data-dependent acquisition (DDA) experiment. The full MS scan was acquired at 70,000 resolution, 106.7–1600 *m*/*z* scan range, 3 × 10^6^ automatic gain control and a 250 ms maximum injection time. From each MS scan, the five ions with the highest intensities were selected for (HR)MS/MS, with a 1.0 Da isolation window, and analyzed at 17,500 resolution, 1 × 10^6^ automatic gain control target, 64 ms max IT, of 37.5 normalized collision energy, 1.5 × 10^5^ intensity threshold and 5 s dynamic exclusion. Total ion chromatographs for representative spectra of each strain are reported in the Supplemental Materials Figures S9–S14.

### 4.4. Processing of the Non-Targeted LC-(HR)MS/MS Dataset

#### 4.4.1. PCA and Factor Loading in R

The raw MS data files collected for the *Planktothrix* intracellular extracts were converted to centroid mzML files using MSConvert v3.1.19 (http://proteowizard.sourceforge.net/, accessed on 22 September 2022) with peak picking set at MS level 1 [47]. Using R 4.1.3 (https://www.r-project.org/, accessed on 22 September 2022), the mzML files were further processed. Feature peak areas were then extracted with the *xcms* package; the parameters are reported in Supplementary Material Table S4. All zero values were imputed with two-thirds of the lowest peak area value measured per metabolite [48]. Deconvolution of the extracted feature peak list involved the removal of features outside the 590–1210 *m*/*z* range, outside the 2.2–5.6 min retention time window, and features from media blanks. Features corresponding to redundant ESI adducts and cyanopeptide isotopic peaks were manually removed. Feature peak areas were multiplied by 50, and peak areas less than the corresponding peak area in the blank were removed. The resulting feature peak area list was log_10_ transformed and Pareto scaled. Using the R packages *MetabolAnalyze* and *FactoMineR*, PCA and factor loading plots were generated to visualize feature variability [49,50]. All dimensions of the PCA were investigated; however, the first and second dimensions were selected for visualization, as they described the most variability between the studied strains. Subsequently, a non-parametric Kruskal–Wallis test with a Benjamini–Hochberg correction was performed to determine the features’ statistical significance and their contribution to the variation within the PCA [51]. Features that possess a *p*-value < 0.01 were deemed significant between strains and are labelled red in the factor loadings plot. Non-significant features appear black, *p*-value > 0.01. An additional factor loading plot was generated, wherein identified cyanopeptide features were color-coded based on their group to show how different cyanopeptide groups drive variation in the PCA. The PCA and factor loading plot data were exported to excel, which allowed selected cyanopeptides to be annotated. Cyanopeptides were identified by comparisons of feature *m*/*z* and RT to available standards or putatively based on interpretation of corresponding MS/MS spectra, literature surveys and database searching: CyanoMetDB and GNPS [11,18]. The plot of cyanopeptide precursor ion *m*/*z* for identified cyanopeptides and product ions diagnostic for each group are reported in Supplemental Materials Figure S2 and Table S3, respectively. The LC-HRMS data for all identified cyanopeptides are tabulated in Supplementary Materials Table S5. A key limitation of HRMS analysis is the inability to distinguish isobaric isomers, including leucine and isoleucine; therefore, they were annotated as ‘Lxx’. New cyanopeptolins detected within this study were named based on their cyanopeptide group, Ahp subclassification and mass. Isobaric cyanopeptolins with identical Ahp feature and mass but different retention times were given trivial names. For example, CP T702A is a cyanopeptolin (CP) with a Thr-Ahp partial sequence (T), a mass of 702.3957 (702) and was the first congener to elute with this mass and Ahp feature (A).

Most cyanopeptides labelled in the loading plot were selected based on volcano plots. Factor loading arrows were labelled based on volcano plot t-test values, wherein the three features with the smallest *t*-test values per strain were labelled; volcano plots are reported in Supplemental Materials Figures S3–S8. Additionally, the three largest arrows from each quadrant were labelled, as a larger magnitude of arrow indicates a stronger influence of that feature on the variance of the dataset. This generated the 30 features that are labelled in the factor loading plots, including 3 aeruginosins, 7 anabaenopeptins, 13 cyanopeptolins, 2 microviridins and 5 features we could not attribute to a cyanopeptide group. Using this strategy, no microcystins were labelled in the loading plots. To generate volcano plots, the deconvoluted HRMS peak list was extracted using the *xcms* R package with peak picking parameters. The volcano plots plot *t*-test (*p*-value < 0.01) and log_2_ values of peak areas of the 154 features.

#### 4.4.2. GNPS Molecular Networking

For molecular networking analysis in GNPS, raw data files generated with the described non-targeted LC-MS/MS experiment were converted to different mzML files using MSConvert, with peak picking set to MS level 1–2. All mzML files were uploaded via FileZilla (https://filezilla-project.org/) to the GNPS network (https://gnps.ucsd.edu/ProteoSAFe/static/gnps-splash.jsp) through the ccms host. The GNPS molecular network was constructed with mzML files for the 48 intracellular *Planktothrix* generated after 14 days, a media blank and seed spectra. Seed spectra included cyanopeptide standards and intracellular extracts from *Microcystis aeruginosa* CPCC 300 and CPCC 632, which were studied by our group for cyanopeptides. MC -LR, [Dha^7^] -LR, -LA and -RR were procured from the National Research Council of Canada’s Metrology Research Centre (Halifax, NS, Canada), the remaining microcystin standards, ferintoic acid A and microginin FR1, were purchased from Enzo Life Sciences, Inc, and cyanopeptolin 982 was purified in-house from *M. aeruginosa* CPCC 464. The molecular network was generated with the following parameters: precursor ion mass tolerance, 0.02 *m*/*z*; product ion mass tolerance, 0.03 *m*/*z*; min pairs cosine score, 0.7; Network TopK, 10; maximum connected component size, 100; minimum matched product ions, 7; minimum cluster size, 2; minimum base peak intensity, 5 × 10^6^; run MSCluster and filter precursor window. From the GNPS website, the graphML file format was uploaded to Cytoscape (https://cytoscape.org/) to visually represent the cluster data. The blank data file was utilized for data deconvolution with the elimination of features present in the blank from the network. Further manual deconvolution of the GNPS molecular network included the removal of features outside 590–1210 *m*/*z*, outside the retention time range 2.2–5.6 min and single nodes unless identified as a cyanopeptide. Features originating from the intracellular *Planktothrix* extracts, seed spectra or shared between the extracts and seed spectra were color coded and annotated based on cyanopeptide group.

## Figures and Tables

**Table 1:** Distribution of all detected cyanopeptides and cyanopeptide groups by studied *Planktothrix* strains.

	*P. rubescens* CPCC 507	*P. agardhii* CPCC 720	*P. rubescens* CPCC 731	*P. rubescens* CPCC 732	*P. rubescens* CPCC 733	*Planktothrix* CPCC 735	Cyanopeptide Group Total
Cyanopeptolins	31	28	17	11	0	23	80
Anabaenopeptins	16	10	14	13	34	12	61
Microviridins	11	17	0	0	14	14	35
Aeruginosins	0	0	19	0	0	9	28
Microcystins	8	4	12	3	6	10	21
Strain total	66	59	62	27	54	68	225

## Data Availability

Additional data will be made available upon request.

## Supplementary Materials

The following supporting information can be downloaded at: https://www.mdpi.com/article/10.3390/toxins16020110/s1, Table S1. Cyanobacteria strain information for studied strains from the Canadian Phycological Culture Collection (CPCC). Table S2. Planktothrix intracellular extract information prior to LC-MS data collection. Table S3: The peak picking parameters used with the bioinformatics R package xcms. Table S4. Cyanopeptide characteristic structural features and the diagnostic product ions used to identify cyanopeptide groups. Table S5. LC-HRMS data for all cyanopeptides detected within *P. rubescens* CPCC 507, P. agardhii CPCC 720, *P. rubescens* CPCC 731, *P. rubescens* CPCC 732, *P. rubescens* CPCC 733, and Planktothrix sp. CPCC 735 intracellular extracts. Figure S1. The six cyanobacterial strains included in our study (left to right): *P. agardhii* CPCC 720, *P. rubescens* CPCC 507, Planktothrix sp. CPCC 735, *P. rubescens* CPCC 732, *P. rubescens* CPCC 731, and *P. rubescens* CPCC 733. Figure S2. Plot of the precursor ion m/z for the 225 cyanopeptides identified within the Planktothrix extracts against their retention time. Figure S3. Volcano plot for *P. rubescens* CPCC 507 features. Figure S4. Volcano plot for *P. agardhii* CPCC 720 features. Figure S5. Volcano plot for *P. rubescens* CPCC 731 features. Figure S6. Volcano plot for *P. rubescens* CPCC 732 features. Figure S7. Volcano plot for *P. rubescens* CPCC 733 features. Figure S8. Volcano plot for Planktothrix sp. CPCC 735 features. Figure S9. TIC for *P. rubescens* CPCC 507. Figure S10. TIC for *P. agardhii* CPCC 720. Figure S11. TIC for *P. rubescens* CPCC 731. Figure S12. TIC for *P. rubescens* CPCC 732. Figure S13. TIC for *P. rubescens* CPCC 733. Figure S14. TIC for *Planktothrix* sp. CPCC 735.
