# Supplementary material for: Cyanopeptolins and Anabaenopeptins Are the Dominant Cyanopeptides from Planktothrix Strains Collected in Canadian Lakes
Source: Toxins (Basel). 2024 Feb 17;16(2):110. doi: 10.3390/toxins16020110 (PMC10891876; doi:10.3390/toxins16020110)
Supplement: Supplementary file 1 [file toxins-16-00110-s001.zip › toxins-2773885-supplementary.pdf]

Supplementary Materials: Cyanopeptolins and Anabaenopeptins are the Dominant Cyanopeptides from *Planktothrix* Strains Collected in Canadian Lakes

Catrina D. Earnshaw and David R. McMullin

Table S1. Cyanobacteria strain information for studied strains from the Canadian Phycological Culture Collection (CPCC).

Strain	Collection Date	Location
P. rubescens CPCC 507	27/28-Jan-1999	Lake Wilcox, ON, Canada
P. agardhii CPCC 720	30-Sep-2007	Brome Lake, QC, Canada
P. rubescens CPCC 731	17-Oct-2008	Vert Lake, QC, Canada
P. rubescens CPCC 732	17-Oct-2008	Vert Lake, QC, Canada
P. rubescens CPCC 733	20-Oct-2008	Vert Lake, QC, Canada
Planktothrix sp. CPCC 735	29-July-2010	Lake Bromont, QC, Canada

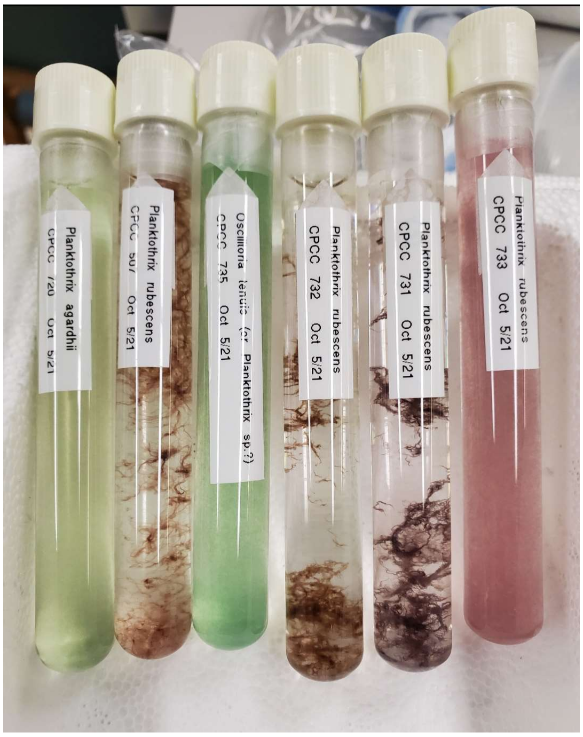

Figure S1. The six cyanobacterial strains included in our study (left to right): *P. agardhii* CPCC 720, *P. rubescens* CPCC 507, *Planktothrix* sp. CPCC 735, *P. rubescens* CPCC 732, *P. rubescens* CPCC 731, and *P. rubescens* CPCC 733.

Table S2. *Planktothrix* intracellular extract information prior to LC-MS data collection.

		Day 14			Day 28		
**Strain**	**Replicate**	**Biomass (mg)**	**Extract (mg)**	**Volume filtered (mL)**	**Biomass (mg)**	**Extract (mg)**	**Volume filtered (mL)**
P. rubescens CPCC 507	1	8.8	1.9	150	20.8	2.3	150
P. rubescens CPCC 507	2	6.2	1.7	150	18.4	2.7	150
P. rubescens CPCC 507	3	6.0	1.8	150	16.6	2.6	150
P. rubescens CPCC 507	4	6.9	5.0	150	12.7	7.8	150
P. agardhii CPCC 720	1	5.4	2.9	100	12.2	6.0	80
P. agardhii CPCC 720	2	7.9	0.7	100	17.8	2.1	80
P. agardhii CPCC 720	3	4.8	1.5	100	9.5	2.8	80
P. agardhii CPCC 720	4	8.1	0.5	100	17.7	2.5	80
P. rubescens CPCC 731	1	8.1	6.4	150	25.7	9.2	150
P. rubescens CPCC 731	2	7.2	4.8	150	24.1	7.0	150
P. rubescens CPCC 731	3	9.3	3.6	150	33.5	6.0	150
P. rubescens CPCC 731	4	8.9	3.5	150	33.0	5.8	150
P. rubescens CPCC 732	1	7.8	1.9	150	23.1	4.6	150
P. rubescens CPCC 732	2	6.2	2.0	150	14.8	5.1	150
P. rubescens CPCC 732	3	8.4	2.5	150	24.3	6.6	150
P. rubescens CPCC 732	4	6.5	1.6	150	16.9	3.8	150
P. rubescens CPCC 733	1	8.9	2.8	100	25.9	6.0	100
P. rubescens CPCC 733	2	10.0	3.5	100	27.1	5.5	100
P. rubescens CPCC 733	3	7.4	2.5	100	23.8	5.0	100
P. rubescens CPCC 733	4	7.6	5.2	100	24.3	12.0	100
Planktothrix sp. CPCC 735	1	4.6	1.2	100	12.0	2.9	70
Planktothrix sp. CPCC 735	2	11.6	1.7	100	30.0	3.8	70
Planktothrix sp. CPCC 735	3	8.0	1.3	100	20.3	6.8	70
Planktothrix sp. CPCC 735	4	10.4	1.1	100	26.4	3.7	70

Table S3. The peak picking parameters used with the bioinformatics R package *xcms*.

Parameter	Polarity	Scan Pre-filter	*m/z* Deviation (ppm)	s/n Ratio cutoff	Peak Width Range (secs)	Noise Level
Set point	Positive	5	1	5	12–15	1×10^6

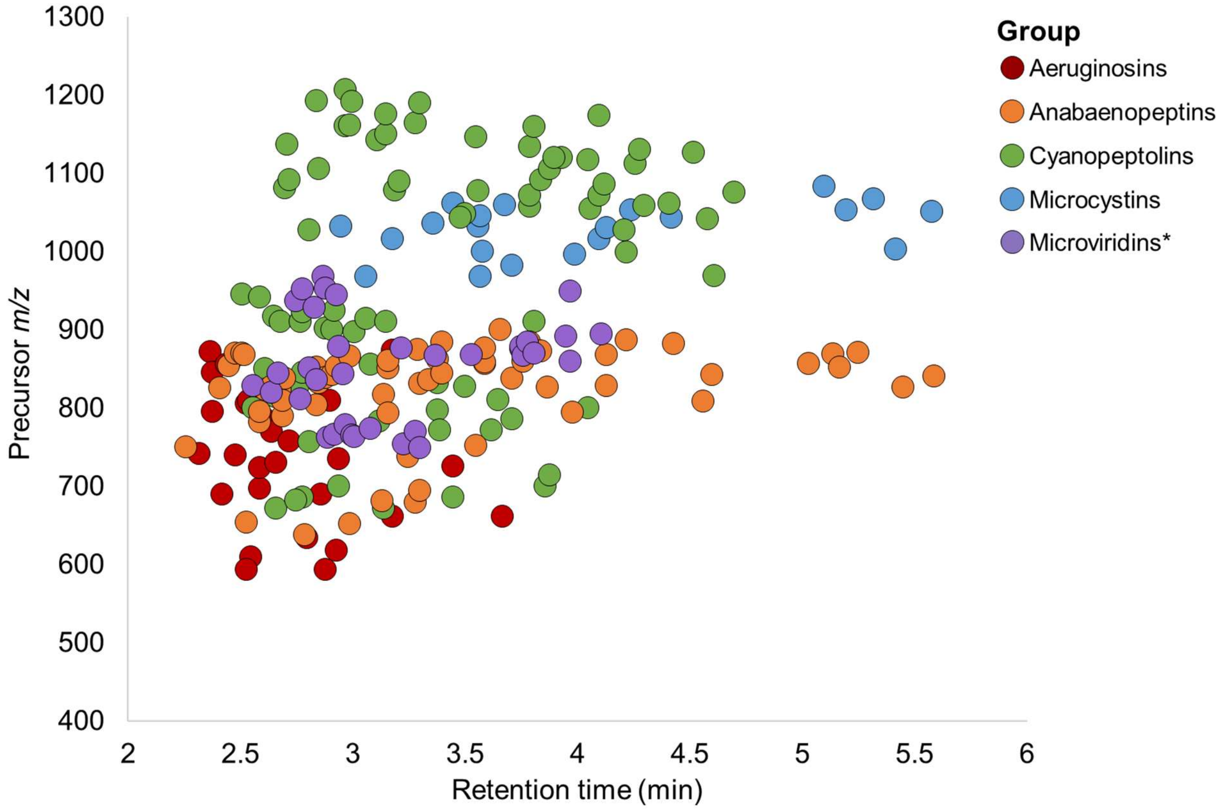

Figure S2. Plot of the precursor ion *m/z* for the 225 cyanopeptides identified within the *Planktothrix* extracts against their retention time. *Indicates $[M+2H]^{2+}$ adduct.

Table S4. Cyanopeptide characteristic structural features and the diagnostic product ions used to identify cyanopeptide groups.

Group	Structural feature(s)	Product ion(s) *m/z*
**Microcystins**	Adda	135.0803, 163.1113
	Mdha	213.0865
**Cyanopeptolins**	Lxx-Ahp	181.1331
	Phe-Ahp	215.1167
	Thr-Ahp	169.0967
	Val-Ahp	167.1541
**Aeruginosins**	Choi	140.1099, 122.0966
	Agma	281.1914
**Microginins**	Di, Mono, Non-Cl-Ahoa	168.0338, 134.0727, 100.1122
	Di, Mono, Non-Cl-*NMe*-Ahoa	182.0494, 148.0883, 114.1278
**Anabaenopeptins**	D-Lys	84.0816, 129.1018
	Arg-CO	201.0985
	Hty	150.09134
**Cyanobactins**	Tzln	86.0059
	MeOx	84.0448
**Microviridins**	Trp	159.0911

Table S5. LC-HRMS data for all cyanopeptides detected within *P. rubescens* CPCC 507, *P. agardhii* CPCC 720, *P. rubescens* CPCC 731, *P. rubescens* CPCC 732, *P. rubescens* CPCC 733, and *Planktothrix* sp. CPCC 735 intracellular extracts.

No	GNPS Cluster #	CNP group	Cyanopeptide	Structural Feature 1	Structural Feature 2	Adduct	*m/z*	Mass Error (ppm)	Chemical formula	RT (min)	507	720	731	732	733	735	Study
1	N/A	MC	MC 1030A	Adda		$[M+H]^+$	1031.5197	0.10	$C_{51}H_{70}N_{10}O_{13}$	2.95	X	X	X				This study
2	N/A	MC	MC 966	Adda		$[M+H]^+$	967.5248	0.10	$C_{47}H_{70}N_{10}O_{12}$	3.06		X	X				This study
3	11	MC	MC 1014A	Adda		$[M+H]^+$	1015.4915	-0.20	$C_{47}H_{70}N_{10}O_{13}S$	3.18			X			X	This study
4	11	MC	MC 1034	Adda		$[M+H]^+$	1035.5510	0.10	$C_{51}H_{74}N_{10}O_{13}$	3.36		X	X				This study
5	11	MC	MC 1061	Adda		$[M+H]^+$	1061.5303	0.09	$C_{52}H_{72}N_{10}O_{14}$	3.45	X		X			X	This study
6	11	MC	MC 1030B	Adda		$[M+H]^+$	1031.5198	0.19	$C_{51}H_{70}N_{10}O_{13}$	3.56	X		X			X	This study
7	11	MC	[Asp^3,Dha^7]-MC-LR	Adda		$[M+H]^+$	967.5250	0.31	$C_{47}H_{70}N_{10}O_{12}$	3.57		X	X			X	McDonald 2023
8	11	MC	[D-Asp^3,(E)-Dhb^7] MC-HtyR	Adda		$[M+H]^+$	1045.5347	-0.57	$C_{52}H_{72}N_{10}O_{13}$	3.57	X		X	X		X	Isaacs 2011
9	11	MC	MC 988	Adda		$[M+H]^+$	999.4966	-0.20	$C_{47}H_{70}N_{10}O_{12}S$	3.58			X			X	This study
10	11	MC	MC 1058	Adda		$[M+H]^+$	1059.5507	-0.28	$C_{53}H_{74}N_{10}O_{13}$	3.68	X						This study
11	11	MC	MC 980	Adda		$[M+H]^+$	981.5390	0.10	$C_{48}H_{72}N_{10}O_{12}$	3.71	X		X	X		X	This study
12	11	MC	MC 994	Adda		$[M+H]^+$	995.5558	-0.20	$C_{49}H_{74}N_{10}O_{12}$	3.99			X	X		X	This study
13	11	MC	MC 1014B	Adda		$[M+H]^+$	1015.5244	-0.30	$C_{51}H_{70}N_{10}O_{12}$	4.10			X			X	This study
14	11	MC	[D-Asp^3,(E)-Dhb^7] MC-HphR	Adda		$[M+H]^+$	1029.5405	0.10	$C_{52}H_{72}N_{10}O_{12}$	4.13	X					X	Isaacs 2011
15	N/A	MC	MC 1051A	Adda		$[M+H]^+$	1052.4974	-0.10	$C_{55}H_{69}N_{7}O_{14}$	4.24					X		This study
16	11	MC	MC 1042	Adda		$[M+H]^+$	1043.5528	-0.19	$C_{53}H_{74}N_{10}O_{12}$	4.42	X						This study
17	12	MC	MC 1081	Adda		$[M+H]^+$	1082.5082	0.09	$C_{56}H_{72}O_{15}N_{7}$	5.10					X		This study
18	12	MC	MC 1051B	Adda		$[M+H]^+$	1052.4976	0.10	$C_{55}H_{69}N_{7}O_{14}$	5.20					X		This study
19	12	MC	MC 1065	Adda		$[M+H]^+$	1066.5131	-0.09	$C_{56}H_{71}N_{7}O_{14}$	5.32					X		This study
20	12	MC	MC-LY	Adda		$[M+H]^+$	1002.5182	-0.10	$C_{52}H_{71}N_{7}O_{13}$	5.42					X		del Campo & Ouahid 2010

21	12	MC	MC 1049	Adda		$[M+H]^+$	1050.5182	-0.10	$C_{56}H_{72}O_{13}N_7$	5.58					X		This study
22	4	AG	AG 740	Choi	Choi-H_2O	$[M+H]^+$	741.3819	0.13	$C_{37}H_{52}O_{10}N_6$	2.32			X				This study
23	4	AG	AG 870	Choi	Choi-H_2O	$[M+H]^+$	871.2941	0.46	$C_{32}H_{52}O_{12}N_{10}Cl_2S$	2.37						X	This study
24	4	AG	AG 874	Choi	Choi-H_2O	$[M+H-SO_3]^+$	795.3921	-0.25	$C_{40}H_{54}O_{14}N_6S$	2.38			X				This study
25	4	AG	AG 844	Choi	Choi-H_2O	$[M+H]^+$	845.3384	-0.24	$C_{39}H_{52}O_{13}N_6S$	2.38			X				This study
26	4	AG	AG 688A	Choi	Choi-H_2O	$[M+H]^+$	689.2962	-0.15	$C_{32}H_{44}N_6O_9S$	2.42						X	McDonald 2023
27	4	AG	AG 854	Choi	Choi-H_2O	$[M+H]^+$	855.2997	0.12	$C_{37}H_{51}O_{13}N_6ClS$	2.44						X	This study
28	4	AG	AG 738	Choi	Choi-H_2O	$[M+H]^+$	739.2524	0.14	$C_{32}H_{43}O_{10}N_6ClS$	2.48						X	This study
29	N/A	AG	AG 672	Choi	Choi-H_2O	$[M+H-SO_3]^+$	593.3448	0.34	$C_{32}H_{44}N_6O_8S$	2.53			X				This study
30	4	AG	AG 804	Choi	Choi-H_2O	$[M+H]^+$	805.3438	0.12	$C_{37}H_{52}O_{12}N_6S$	2.53			X				This study
31	4	AG	AG 888A	Choi	Choi-H_2O	$[M+H-SO_3]^+$	809.3445	0.12	$C_{42}H_{60}O_{13}N_6S$	2.54						X	This study
32	4	AG	AG 608	Choi	Choi-H_2O	$[M+H]^+$	609.3391	-0.66	$C_{32}H_{45}O_6N_6$	2.55			X				This study
33	4	AG	AG 696	Choi	Choi-H_2O	$[M+H]^+$	697.3013	-0.14	$C_{34}H_{44}O_8N_6S$	2.59			X				This study
34	4	AG	AG 722	Choi	Choi-H_2O	$[M+H]^+$	723.2575	0.14	$C_{32}H_{43}N_6O_9ClS$	2.59						X	McDonald 2023
35	4	AG	AG 787	Choi	Choi-H_2O	$[M+H]^+$	788.3990	-0.13	$C_{42}H_{50}O_5N_{11}$	2.60			X				This study
36	4	AG	AG 828	Choi	Choi-H_2O	$[M+H]^+$	829.3435	-0.24	$C_{39}H_{52}O_{12}N_6S$	2.60			X				This study
37	4	AG	AG 768	Choi	Choi-H_2O	$[M+H]^+$	769.2047	-0.13	$C_{37}H_{29}O_6N_{12}S$	2.64						X	This study
38	4	AG	AG 728	Choi	Choi-H_2O	$[M+H]^+$	729.4187	0.69	$C_{37}H_{56}O_9N_6$	2.66			X				This study
39	4	AG	AG 756	Choi	Choi-H_2O	$[M+H]^+$	757.2180	-0.40	$C_{32}H_{42}N_6O_9Cl_2S$	2.72						X	This study
40	4	AG	AG 632	Choi	Choi-H_2O	$[M+H]^+$	633.3394	-0.16	$C_{34}H_{44}O_6N_6$	2.80			X				This study
41	N/A	AG	AG 688B	Choi	Choi-H_2O	$[M+H]^+$	689.3425	0.15	$C_{23}H_{49}O_{14}N_{10}$	2.86			X				This study
42	4	AG	AG 592	Choi	Choi-H_2O	$[M+H]^+$	593.3447	0.17	$C_{32}H_{44}N_6O_5$	2.88			X				This study
43	4	AG	AG 888B	Choi	Choi-H_2O	$[M+H-SO_3]^+$	809.4445	0.25	$C_{42}H_{60}O_{13}N_6S$	2.90			X				This study

44	4	AG	AG A	Choi	Choi-H_2O	$[M+H]^+$	617.3448	0.32	$C_{34}H_{44}N_6O_5$	2.93			X				Cy-anoMetDB 2023
45	4	AG	AG 733	Choi	Choi-H_2O	$[M+H]^+$	734.4239	0.41	$C_{39}H_{55}O_7N_7$	2.94			X				This study
46	N/A	AG	AG 660A	Choi	Choi-H_2O	$[M+H]^+$	661.4070	-0.30	$C_{37}H_{52}O_5N_6$	3.18			X				This study
47	4	AG	AG 872	Choi	Choi-H_2O	$[M+H]^+$	873.4064	0.11	$C_{42}H_{60}O_{12}N_6S$	3.18			X				This study
48	4	AG	AG 724	Choi	Choi-H_2O	$[M+H]^+$	725.3851	0.14	$C_{49}H_{49}O_2N_4$	3.45						X	This study
49	4	AG	AG 660B	Choi	Choi-H_2O	$[M+H]^+$	661.4069	-0.45	$C_{37}H_{52}O_5N_6$	3.67			X				This study
50	3	CP	CP T864	$[Thr\text{-}Ahp–H_2O–CO+H]^+$	$[Thr\text{-}Ahp–H_2O+H]^+$	$[M+H]^+$	865.4134	0.35	$C_{47}H_{56}O_{10}N_6$	2.48						X	This study
51	3	CP	CP T944	$[Thr\text{-}Ahp–H_2O–CO+H]^+$	$[Thr\text{-}Ahp–H_2O+H]^+$	$[M+H]^+$	945.5039	-0.11	$C_{44}H_{68}O_{13}N_{10}$	2.51						X	This study
52	3	CP	CP T816A	$[Thr\text{-}Ahp–H_2O–CO+H]^+$	$[Thr\text{-}Ahp–H_2O+H]^+$	$[M+H\text{-}H_2O]^+$	799.4348	-0.13	$C_{39}H_{60}O_{11}N_8$	2.56	X	X				X	This study
53	3	CP	CP T958	$[Thr\text{-}Ahp–H_2O–CO+H]^+$	$[Thr\text{-}Ahp–H_2O+H]^+$	$[M+H\text{-}H_2O]^+$	941.5082	0.42	$C_{44}H_{74}O_{17}N_6$	2.59						X	This study
54	3	CP	CP T848	$[Thr\text{-}Ahp–H_2O–CO+H]^+$	$[Thr\text{-}Ahp–H_2O+H]^+$	$[M+H]^+$	849.4159	-0.12	$C_{30}H_{62}O_{18}N_{10}$	2.61						X	This study
55	3	CP	CP T915	$[Thr\text{-}Ahp–H_2O–CO+H]^+$	$[Thr\text{-}Ahp–H_2O+H]^+$	$[M+H]^+$	916.5135	-0.33	$C_{44}H_{69}O_{12}N_9$	2.65	X						This study
56	3	CP	CP T688A	$[Thr\text{-}Ahp–H_2O–CO+H]^+$	$[Thr\text{-}Ahp–H_2O+H]^+$	$[M+H\text{-}H_2O]^+$	671.3765	0.30	$C_{34}H_{52}O_9N_6$	2.66		X					This study
57	3	CP	CP T830A	$[Thr\text{-}Ahp–H_2O–CO+H]^+$	$[Thr\text{-}Ahp–H_2O+H]^+$	$[M+H\text{-}H_2O]^+$	813.4503	-0.25	$C_{40}H_{62}O_{11}N_8$	2.66	X	X				X	This study

58	N/A	CP	CP T927A	[Thr-Ahp–H_2O–CO+H]$^+$	[Thr-Ahp–H_2O+H]$^+$	[M+H-H_2O]$^+$	910.5018	-0.11	$C_{44}H_{73}O_{16}N_5$	2.68	X	X					This study
59	3	CP	CP T1079	[Thr-Ahp–H_2O–CO+H]$^+$	[Thr-Ahp–H_2O+H]$^+$	[M+H]$^+$	1080.5229	-0.09	$C_{64}H_{69}O_9N_7$	2.70		X					This study
60	3	CP	CP T1135	[Thr-Ahp–H_2O–CO+H]$^+$	[Thr-Ahp–H_2O+H]$^+$	[M+H]$^+$	1136.5985	-0.09	$C_{55}H_{81}O_{15}N_{11}$	2.71						X	This study
61	3	CP	CP T1090	[Thr-Ahp–H_2O–CO+H]$^+$	[Thr-Ahp–H_2O+H]$^+$	[M+H]$^+$	1091.5759	0.09	$C_{53}H_{82}O_{18}N_6$	2.72	X						This study
62	3	CP	CP T844A	[Thr-Ahp–H_2O–CO+H]$^+$	[Thr-Ahp–H_2O+H]$^+$	[M+H-H_2O]$^+$	827.4663	0.12	$C_{41}H_{64}O_{11}N_8$	2.76	X					X	This study
63	3	CP	CP T927B	[Thr-Ahp–H2O–CO+H]$^+$	[Thr-Ahp–H_2O+H]$^+$	[M+H-H_2O]$^+$	910.4667	-0.21	$C_{44}H_{65}O_{13}N_9$	2.77						X	This study
64	3	CP	CP T702A	[Thr-Ahp–H_2O–CO+H]$^+$	[Thr-Ahp–H_2O+H]$^+$	[M+H-H_2O]$^+$	685.3918	-0.15	$C_{35}H_{54}O_9N_6$	2.78	X	X				X	This study
65	Ind. Node	CP	CP T939	[Thr-Ahp–H_2O–CO+H]$^+$	[Thr-Ahp–H_2O+H]$^+$	[M+H-H_2O]$^+$	922.5018	-0.11	$C_{45}H_{73}O_{16}N_5$	2.78	X						This study
66	3	CP	CP T1044	[Thr-Ahp–H_2O–CO+H]$^+$	[Thr-Ahp–H_2O+H]$^+$	[M+H-H_2O]$^+$	1027.5296	0.10	$C_{48}H_{72}O_{16}N_{10}$	2.81						X	This study
67	3	CP	CP T773	[Thr-Ahp–H_2O–CO+H]$^+$	[Thr-Ahp–H_2O+H]$^+$	[M+H-H_2O]$^+$	756.4292	0.13	$C_{38}H_{59}O_{10}N_7$	2.81	X						This study
68	3	CP	CP T1209	[Thr-Ahp–H_2O–CO+H]$^+$	[Thr-Ahp–H_2O+H]$^+$	[M+H-H_2O]$^+$	1192.5886	0.08	$C_{57}H_{83}O_{18}N_{11}$	2.84						X	This study

69	3	CP	CP T1104	[Thr-Ahp–H_2O–CO+H]$^+$	[Thr-Ahp–H_2O+H]$^+$	[M+H]$^+$	1105.5929	0.09	$C_{55}H_{81}O_{14}N_{10}$	2.85	X	X					This study
70	3	CP	CP T918	[Thr-Ahp–H_2O–CO+H]$^+$	[Thr-Ahp–H_2O+H]$^+$	[M+H-H_2O]$^+$	901.4665	-0.11	$C_{43}H_{66}O_{14}N_8$	2.88	X						This study
71	3	CP	CP T916	[Thr-Ahp–H_2O–CO+H]$^+$	[Thr-Ahp–H_2O+H]$^+$	[M+H-H_2O]$^+$	899.4507	-0.22	$C_{43}H_{64}O_{14}N_8$	2.91	X	X				X	This study
72	3	CP	CP T941	[Thr-Ahp–H_2O–CO+H]$^+$	[Thr-Ahp–H_2O+H]$^+$	[M+H-H_2O]$^+$	924.4822	-0.32	$C_{45}H_{67}O_{13}N_9$	2.92						X	This study
73	3	CP	CP T716A	[Thr-Ahp–H_2O–CO+H]$^+$	[Thr-Ahp–H_2O+H]$^+$	[M+H-H_2O]$^+$	699.4075	-0.14	$C_{36}H_{56}O_9N_6$	2.94		X					This study
74	3	CP	CP T1177	[Thr-Ahp–H_2O–CO+H]$^+$	[Thr-Ahp–H_2O+H]$^+$	[M+H-H_2O]$^+$	1160.5664	0.09	$C_{61}H_{79}O_{15}N_9$	2.97						X	This study
75	3	CP	CP T1223	[Thr-Ahp–H_2O–CO+H]$^+$	[Thr-Ahp–H_2O+H]$^+$	[M+H-H_2O]$^+$	1206.6038	-0.24	$C_{58}H_{85}O_{18}N_{11}$	2.97						X	This study
76	3	CP	CP T1178	[Thr-Ahp–H_2O–CO+H]$^+$	[Thr-Ahp–H_2O+H]$^+$	[M+H-H_2O]$^+$	1161.5826	-0.09	$C_{57}H_{82}O_{17}N_{10}$	2.99	X	X					This study
77	3	CP	CP T1208	[Thr-Ahp–H_2O–CO+H]$^+$	[Thr-Ahp–H_2O+H]$^+$	[M+H-H_2O]$^+$	1191.5933	0.08	$C_{58}H_{84}O_{18}N_{10}$	3.00	X						This study
78	3	CP	CP T913	[Thr-Ahp–H_2O–CO+H]$^+$	[Thr-Ahp–H_2O+H]$^+$	[M+H-H_2O]$^+$	896.4865	-0.11	$C_{44}H_{67}O_{12}N_9$	3.01	X	X					This study
79	3	CP	CP T930	[Thr-Ahp–H_2O–CO+H]$^+$	[Thr-Ahp–H_2O+H]$^+$	[M+H-H_2O]$^+$	913.4662	-0.33	$C_{44}H_{66}O_{14}N_8$	3.06		X					This study

80	3	CP	CP T872	[Thr-Ahp–H_2O–CO+H]$^+$	[Thr-Ahp–H_2O+H]$^+$	[M+H-H_2O]$^+$	855.4609	-0.23	$C_{42}H_{64}O_{12}N_8$	3.08	X						This study
81	3	CP	CP T1159	[Thr-Ahp–H_2O–CO+H]$^+$	[Thr-Ahp–H_2O+H]$^+$	[M+H-H_2O]$^+$	1142.6095	0.26	$C_{54}H_{85}O_{17}N_{11}$	3.11						X	This study
82	3	CP	CP T799	[Thr-Ahp–H_2O–CO+H]$^+$	[Thr-Ahp–H_2O+H]$^+$	[M+H-H_2O]$^+$	782.4081	-0.26	$C_{39}H_{57}O_{11}N_7$	3.12		X				X	This study
83	3	CP	CP T688B	[Thr-Ahp–H_2O–CO+H]$^+$	[Thr-Ahp–H_2O+H]$^+$	[M+H-H_2O]$^+$	671.3762	-0.14	$C_{34}H_{52}O_9N_6$	3.14		X					This study
84	3	CP	CP T1166	[Thr-Ahp–H_2O–CO+H]$^+$	[Thr-Ahp–H_2O+H]$^+$	[M+H-H_2O]$^+$	1149.5828	0.09	$C_{56}H_{82}O_{17}N_{10}$	3.15	X						This study
85	3	CP	CP T1192	[Thr-Ahp–H_2O–CO+H]$^+$	[Thr-Ahp–H_2O+H]$^+$	[M+H-H_2O]$^+$	1175.5964	-0.09	$C_{70}H_{80}O_{10}N_8$	3.15	X	X					This study
86	3	CP	CP T927C	[Thr-Ahp–H_2O–CO+H]$^+$	[Thr-Ahp–H_2O+H]$^+$	[M+H-H_2O]$^+$	910.5030	-0.33	$C_{45}H_{69}O_{12}N_9$	3.15	X	X					This study
87	3	CP	CP T1095	[Thr-Ahp–H_2O–CO+H]$^+$	[Thr-Ahp–H_2O+H]$^+$	[M+H-H_2O]$^+$	1078.5436	-0.09	$C_{65}H_{73}O_9N_7$	3.19	X					X	This study
88	3	CP	CP T1088	[Thr-Ahp–H_2O–CO+H]$^+$	[Thr-Ahp–H_2O+H]$^+$	[M+H]$^+$	1089.5967	0.09	$C_{54}H_{84}O_{17}N_6$	3.21	X						This study
89	3	CP	CP T1180	[Thr-Ahp–H_2O–CO+H]$^+$	[Thr-Ahp–H_2O+H]$^+$	[M+H-H_2O]$^+$	1163.5985	0.17	$C_{57}H_{84}O_{17}N_{10}$	3.28	X						This study
90	3	CP	CP T1206	[Thr-Ahp–H_2O–CO+H]$^+$	[Thr-Ahp–H_2O+H]$^+$	[M+H-H_2O]$^+$	1189.6139	-0.08	$C_{59}H_{86}O_{17}N_{10}$	3.30		X					This study

91	3	CP	CP T813	[Thr-Ahp–H_2O–CO+H]$^+$	[Thr-Ahp–H_2O+H]$^+$	[M+H-H_2O]$^+$	796.4238	-0.25	$C_{40}H_{59}O_{11}N_7$	3.38	X	X				X	This study
92	3	CP	CP T830B	[Thr-Ahp–H_2O–CO+H]$^+$	[Thr-Ahp–H_2O+H]$^+$	[M+H]$^+$	831.4612	0.12	$C_{40}H_{62}O_{11}N_8$	3.38		X					This study
93	N/A	CP	CP T788A	[Thr-Ahp–H_2O–CO+H]$^+$	[Thr-Ahp–H_2O+H]$^+$	[M+H-H_2O]$^+$	771.3920	-0.39	$C_{38}H_{56}O_{12}N_6$	3.39		X					This study
94	3	CP	CP T702B	[Thr-Ahp–H_2O–CO+H]$^+$	[Thr-Ahp–H_2O+H]$^+$	[M+H-H_2O]$^+$	685.3920	0.15	$C_{35}H_{54}O_9N_6$	3.45		X				X	This study
95	Ind. Node	CP	CP T1064	[Thr-Ahp–H_2O–CO+H]$^+$	[Thr-Ahp–H_2O+H]$^+$	[M+H-H_2O]$^+$	1047.5394	-0.29	$C_{53}H_{76}O_{15}N_8$	3.50	X						This study
96	3	CP	CP T844B	[Thr-Ahp–H_2O–CO+H]$^+$	[Thr-Ahp–H_2O+H]$^+$	[M+H-H_2O]$^+$	827.4297	-0.12	$C_{40}H_{60}O_{12}N_8$	3.50						X	This study
97	3	CP	CP T1162	[Thr-Ahp–H_2O–CO+H]$^+$	[Thr-Ahp–H_2O+H]$^+$	[M+H-H_2O]$^+$	1145.5861	0.17	$C_{69}H_{78}O_9N_8$	3.55	X						This study
98	3	CP	CP T788B	[Thr-Ahp–H_2O–CO+H]$^+$	[Thr-Ahp–H_2O+H]$^+$	[M+H-H_2O]$^+$	771.3922	-0.12	$C_{38}H_{56}O_{12}N_6$	3.62	X	X				X	This study
99	3	CP	CP T827	[Thr-Ahp–H_2O–CO+H]$^+$	[Thr-Ahp–H_2O+H]$^+$	[M+H-H_2O]$^+$	810.4395	-0.12	$C_{41}H_{61}O_{11}N_7$	3.65		X					This study
100	3	CP	CP T802	[Thr-Ahp–H_2O–CO+H]$^+$	[Thr-Ahp–H_2O+H]$^+$	[M+H-H_2O]$^+$	785.4081	0.13	$C_{39}H_{58}O_{12}N_6$	3.71		X					This study
101	3	CP	CP T1150	[Thr-Ahp–H_2O–CO+H]$^+$	[Thr-Ahp–H_2O+H]$^+$	[M+H-H_2O]$^+$	1133.5863	-0.09	$C_{55}H_{86}O_{20}N_6$	3.79	X						This study

102	3	CP	CP T1176	[Thr-Ahp–H_2O–CO+H]$^+$	[Thr-Ahp–H_2O+H]$^+$	[M+H-H_2O]$^+$	1159.6023	0.17	$C_{57}H_{88}O_{20}N_6$	3.81	X	X					This study
103	N/A	CP	CP T927D	[Thr-Ahp–H_2O–CO+H]$^+$	[Thr-Ahp–H_2O+H]$^+$	[M+H-H_2O]$^+$	910.5032	-0.11	$C_{45}H_{69}O_{12}N_9$	3.81	X						This study
104	N/A	CP	CP T716B	[Thr-Ahp–H_2O–CO+H]$^+$	[Thr-Ahp–H_2O+H]$^+$	[M+H-H_2O]$^+$	699.4075	-0.14	$C_{36}H_{56}O_9N_6$	3.86		X					This study
105	3	CP	CP T730	[Thr-Ahp–H_2O–CO+H]$^+$	[Thr-Ahp–H_2O+H]$^+$	[M+H-H_2O]$^+$	713.3870	0.14	$C_{36}H_{54}O_{10}N_6$	3.88		X					This study
106	N/A	CP	CP T816B	[Thr-Ahp–H_2O–CO+H]$^+$	[Thr-Ahp–H_2O+H]$^+$	[M+H-H_2O]$^+$	799.4235	-0.12	$C_{40}H_{60}O_{12}N_6$	4.05		X					This study
107	3	CP	CP T1190	[Thr-Ahp–H_2O–CO+H]$^+$	[Thr-Ahp–H_2O+H]$^+$	[M+H-H_2O]$^+$	1173.6189	-0.09	$C_{59}H_{86}O_{16}N_{10}$	4.10	X						This study
108	3	CP	CP T1075	[Thr-Ahp–H_2O–CO+H]$^+$	[Thr-Ahp–H_2O+H]$^+$	[M+H-H_2O]$^+$	1058.5558	0.09	$C_{54}H_{77}O_{14}N_9$	4.30	X						This study
109	N/A	CP	CP P680	[Phe-Ahp–H_2O–CO+H]$^+$	[Phe-Ahp–H_2O+H]$^+$	[M+H]$^+$	681.3972	0.29	$C_{36}H_{52}O_7N_6$	2.75			X				This study
110	1	CP	CP L1060	[Lxx-Ahp–H_2O–CO+H]$^+$	[Lxx-Ahp–H_2O+H]$^+$	[M+H-H_2O]$^+$	1043.4752	-0.19	$C_{49}H_{72}O_{16}N_8S$	3.48			X	X			This study
111	1	CP	CP P1094	[Phe-Ahp–H_2O–CO+H]$^+$	[Phe-Ahp–H_2O+H]$^+$	[M+H-H_2O]$^+$	1077.4578	-0.09	$C_{64}H_{66}O_9N_6S$	3.56			X	X			This study
112	2	CP	CP L1074	[Lxx-Ahp–H_2O–CO+H]$^+$	[Lxx-Ahp–H_2O+H]$^+$	[M+H-H_2O]$^+$	1057.4897	0.47	$C_{62}H_{70}O_9N_6S$	3.79			X	X			This study

113	1	CP	CP L1088	[Lxx-Ahp–H_2O–CO+H]$^+$	[Lxx-Ahp–H_2O+H]$^+$	[M+H-H_2O]$^+$	1071.5065	-0.19	$C_{51}H_{76}N_8O_{16}S$	3.79				X			This study
114	1	CP	CP P1108	[Phe-Ahp–H_2O–CO+H]$^+$	[Phe-Ahp–H_2O+H]$^+$	[M+H-H_2O]$^+$	1091.4738	0.18	$C_{65}H_{68}O_9N_6S$	3.84				X			This study
115	1	CP	CP P1122	[Phe-Ahp–H_2O–CO+H]$^+$	[Phe-Ahp–H_2O+H]$^+$	[M+H-H_2O]$^+$	1105.4896	0.36	$C_{66}H_{70}O_9N_6S$	3.88			X	X			This study
116	2	CP	CP P1136	[Phe-Ahp–H_2O–CO+H]$^+$	[Phe-Ahp–H_2O+H]$^+$	[M+H-H_2O]$^+$	1119.5065	-0.18	$C_{55}H_{76}O_{16}N_8S$	3.90			X	X			This study
117	2	CP	CP P1115	[Phe-Ahp–H_2O–CO+H]$^+$	[Phe-Ahp–H_2O+H]$^+$	[M+H]$^+$	1116.4595	0.09	$C_{55}H_{69}O_{16}N_7S$	4.05			X				This study
118	Ind. Node	CP	CP P1053	[Phe-Ahp–H_2O–CO+H]$^+$	[Phe-Ahp–H_2O+H]$^+$	[M+H]$^+$	1054.5359	0.28	$C_{53}H_{71}O_{12}N_{11}$	4.06			X				This study
119	1	CP	CP V1088	[Val-Ahp–H_2O–CO+H]$^+$	[Val-Ahp–H2O+H]$^+$	[M+H-H_2O]$^+$	1071.5065	-0.18	$C_{51}H_{76}O_{16}N_8S$	4.10			X	X			This study
120	1	CP	CP V1102	[Val-Ahp–H_2O–CO+H]$^+$	[Val-Ahp–H2O+H]$^+$	[M+H-H_2O]$^+$	1085.5223	-0.09	$C_{52}H_{78}O_{16}N_8S$	4.12				X			This study
121	1	CP	CP P1044	[Phe-Ahp–H_2O–CO+H]$^+$	[Phe-Ahp–H_2O+H]$^+$	[M+H-H_2O]$^+$	1027.4806	0.10	$C_{49}H_{72}O_{15}N_8S$	4.21			X				This study
122	Ind. Node	CP	CP L1015	[Lxx-Ahp–H_2O–CO+H]$^+$	[Lxx-Ahp–H_2O+H]$^+$	[M+H-H_2O]$^+$	998.5234	0.10	$C_{53}H_{73}O_{13}N_7$	4.22			X				This study
123	2	CP	CP P1129	[Phe-Ahp–H_2O–CO+H]$^+$	[Phe-Ahp–H_2O+H]$^+$	[M+H-H_2O]$^+$	1112.4644	-0.09	$C_{56}H_{71}O_{16}N_7S$	4.26			X				This study

124	2	CP	CP P1147	[Phe-Ahp–H_2O–CO+H]$^+$	[Phe-Ahp–H_2O+H]$^+$	[M+H-H_2O]$^+$	1130.4752	0.09	$C_{56}H_{73}O_{17}N_7S$	4.28			X				This study
125	1	CP	CP P1078	[Phe-Ahp–H_2O–CO+H]$^+$	[Phe-Ahp–H_2O+H]$^+$	[M+H-H_2O]$^+$	1061.4647	-0.09	$C_{52}H_{70}O_{15}N_8S$	4.41			X				This study
126	2	CP	CP P1143	[Phe-Ahp–H_2O–CO+H]$^+$	[Phe-Ahp–H_2O+H]$^+$	[M+H-H_2O]$^+$	1126.4445	0.62	$C_{56}H_{69}O_{17}N_7S$	4.52				X			This study
127	1	CP	CP P1058	[Phe-Ahp–H_2O–CO+H]$^+$	[Phe-Ahp–H_2O+H]$^+$	[M+H-H_2O]$^+$	1041.4966	0.48	$C_{50}H_{74}O_{15}N_8S$	4.58			X				This study
128	2	CP	CP P985	[Phe-Ahp–H_2O–CO+H]$^+$	[Phe-Ahp–H_2O+H]$^+$	[M+H-H_2O]$^+$	968.5142	0.10	$C_{53}H_{67}O_8N_{11}$	4.61			X				This study
129	1	CP	CP P1092	[Phe-Ahp–H_2O–CO+H]$^+$	[Phe-Ahp–H_2O+H]$^+$	[M+H-H_2O]$^+$	1075.4810	0.46	$C_{53}H_{72}O_{15}N_8S$	4.70			X	X			This study
130	6	AP	AP 748	Lys	Hty	[M+H]$^+$	749.4190	-0.27	$C_{35}H_{56}O_{10}N_8$	2.26					X		This study
131	6	AP	AP 824	Lys	Phe	[M+H]$^+$	825.4504	-0.12	$C_{41}H_{60}O_{10}N_8$	2.41					X		This study
132	5	AP	AP 852	Lys	Arg	[M+H]$^+$	853.4565	-0.12	$C_{41}H_{60}O_{10}N_{10}$	2.45					X		This study
133	5	AP	AP 868A	Lys	Arg	[M+H]$^+$	869.4879	-0.12	$C_{42}H_{64}O_{10}N_{10}$	2.48			X				This study
134	N/A	AP	AP 868B	Lys	Arg	[M+H]$^+$	869.3676	0.12	$C_{40}H_{53}O_{14}N_8$	2.51			X				This study
135	5	AP	AP 866	Lys	Arg	[M+H]$^+$	867.4926	0.35	$C_{42}H_{62}O_{10}N_{10}$	2.52			X	X			This study
136	6	AP	AP 652	Lys	Phe	[M+H]$^+$	653.3658	0.15	$C_{34}H_{49}O_7N_6$	2.53					X		This study
137	Ind. Node	AP	AP 780	Lys	Phe	[M+H]$^+$	781.4244	0.13	$C_{39}H_{56}O_9N_8$	2.59					X		This study
138	6	AP	AP 794	Lys	Phe	[M+H]$^+$	795.4401	0.13	$C_{40}H_{58}O_9N_8$	2.59					X		This study
139	5	AP	AP 822A	Lys	Arg	[M+H]$^+$	823.4449	0.12	$C_{39}H_{62}O_{13}N_6$	2.61					X		This study
140	6	AP	AP 826	Lys	Phe	[M+H]$^+$	827.4122	-0.48	$C_{48}H_{54}O_7N_6$	2.64					X		This study
141	6	AP	AP 822B	Lys	Phe	[M+H]$^+$	823.4715	0.36	$C_{42}H_{63}O_9N_8$	2.68		X	X	X	X		This study
142	6	AP	AP 788	Lys	Hty	[M+H]$^+$	789.4870	0.13	$C_{39}H_{64}O_9N_8$	2.69				X			This study
143	6	AP	AP 808	Lys	Phe	[M+H]$^+$	809.4553	-0.37	$C_{41}H_{60}O_9N_8$	2.69		X	X	X	X		This study

144	5	AP	AP B	Lys	Arg	[M+H]+	837.4617	-0.12	$C_{41}H_{60}N_{10}O_{9}$	2.70		X	X	X	X	X	Harada et al. 1995
145	6	AP	AP 636	Lys	Trp	[M+H]+	637.3707	-0.16	$C_{34}H_{48}O_{6}N_{6}$	2.79	X	X	X		X	X	This study
146	6	AP	AP 802	Lys	Trp	[M+H]+	803.5023	-0.25	$C_{40}H_{66}O_{9}N_{8}$	2.84				X			This study
147	5	AP	AP E	Lys	Arg	[M+H]+	851.4771	-0.35	$C_{42}H_{62}N_{10}O_{9}$	2.84		X	X	X	X		Shin et al. 1998
148	5	AP	AP 830	Lys	Arg	[M+H]+	831.5090	0.36	$C_{40}H_{66}O_{9}N_{10}$	2.85			X	X			This study
149	Ind. node	AP	AP 837	Lys	Arg	[M+H]+	838.4420	0.36	$C_{36}H_{59}O_{12}N_{11}$	2.88		X					This study
150	6	AP	AP 841A	Lys	Phe	[M+H]+	842.4114	-0.12	$C_{33}H_{55}O_{13}N_{13}$	2.91	X						This study
151	6	AP	AP 851	Lys	Arg	[M+H]+	852.4576	0.23	$C_{37}H_{61}O_{12}N_{11}$	2.93					X		This study
152	6	AP	AP 650	Lys	Phe	[M+H]+	651.3865	0.15	$C_{35}H_{50}O_{6}N_{6}$	2.99	X		X	X	X	X	This study
153	5	AP	AP 864	Lys	Arg	[M+H]+	865.4936	0.58	$C_{43}H_{64}O_{9}N_{10}$	2.99			X	X			This study
154	6	AP	AP 679	Lys	Phe	[M+H]+	680.3767	0.15	$C_{35}H_{49}O_{7}N_{7}$	3.13		X			X		Harms et al. 2016
155	6	AP	AP 815	Lys	Phe	[M+H]+	816.3926	-0.12	$C_{42}H_{53}O_{10}N_{7}$	3.14					X		This study
156	6	AP	AP 792	Lys	Phe	[M+H]+	793.4603	-0.38	$C_{41}H_{60}O_{8}N_{8}$	3.16					X		This study
157	6	AP	AP 849	Lys	Phe	[M+H]+	850.4708	-0.12	$C_{44}H_{63}O_{10}N_{7}$	3.16					X		This study
158	6	AP	AP 859	Lys	Phe	[M+H]+	860.4193	0.46	$C_{44}H_{57}O_{11}N_{7}$	3.16					X		This study
159	6	AP	AP 736	Lys	Phe	[M+H]+	737.3870	0.14	$C_{38}H_{52}O_{9}N_{6}$	3.25		X			X	X	This study
160	6	AP	AP 678	Lys	Phe	[M+H]+	679.3813	-0.15	$C_{36}H_{50}O_{7}N_{6}$	3.28					X		This study
161	6	AP	AP 873	Lys	Trp	[M+H]+	874.3982	0.11	$C_{44}H_{55}O_{12}N_{7}$	3.29					X		This study
162	6	AP	AP 693	Lys	Phe	[M+H]+	694.3922	-0.14	$C_{36}H_{51}O_{7}N_{7}$	3.30					X		This study
163	6	AP	AP 829	Lys	Phe	[M+H]+	830.4084	0.12	$C_{43}H_{55}O_{10}N_{7}$	3.30					X		This study
164	5	AP	AP 834	Lys	Arg	[M+H]+	835.4821	-0.48	$C_{42}H_{62}O_{8}N_{10}$	3.34			X	X			This study
165	6	AP	AP 861	Lys	Phe	[M+H]+	862.3782	-0.23	$C_{47}H_{47}O_{6}N_{11}$	3.38					X		This study
166	6	AP	AP 882A	Lys	Phe	[M+H]+	883.4351	0.23	$C_{46}H_{58}O_{10}N_{8}$	3.40						X	McDonald 2023
167	6	AP	AP A	Lys	Phe	[M+H]+	844.4238	-0.24	$C_{44}H_{57}N_{7}O_{10}$	3.40	X	X			X	X	Harada et al. 1995
168	6	AP	AP 750	Lys	Phe	[M+H]+	751.4026	0.13	$C_{39}H_{54}O_{9}N_{6}$	3.55			X	X			This study
169	6	AP	AP 855	Lys	Phe	[M+H]+	856.4236	-0.47	$C_{45}H_{57}O_{10}N_{7}$	3.59					X		This study

170	6	AP	AP 857	Lys	Trp	$[M+H]^+$	858.4392	-0.47	$C_{45}H_{59}N_7O_{10}$	3.59	X				X		McDonald 2023
171	6	AP	AP 875	Lys	Trp	$[M+H]^+$	876.3860	0.11	$C_{36}H_{53}O_{13}N_{13}$	3.59					X		This study
172	6	AP	AP 898	Lys	Phe	$[M+H]^+$	899.4274	0.33	$C_{42}H_{54}O_9N_{14}$	3.66						X	This study
173	6	AP	AP 836	Lys	Arg-CO	$[M+H]^+$	837.3817	-0.12	$C_{45}H_{52}O_{10}N_6$	3.71					X		This study
174	6	AP	AP 858	Lys	Trp	$[M+H]^+$	859.3761	0.11	$C_{44}H_{49}O_8N_{11}$	3.76					X		This study
175	N/A	AP	AP 882B	Lys	Trp	$[M+H]^+$	883.4352	0.34	$C_{46}H_{58}N_8O_{10}$	3.79						X	This study
176	6	AP	AP 872	Lys	2-amino-5-hydroxy-phenylpentanoic acid	$[M+H]^+$	872.4547	-0.57	$C_{46}H_{61}N_7O_{10}$	3.84	X						Isaacs 2011
177	N/A	AP	AP 825A	Lys		$[M+H]^+$	826.4167	0.12	$C_{33}H_{56}O_{12}N_{13}$	3.87	X						This study
178	6	AP	AP J	Lys	Phe	$[M+H]^+$	794.4445	-0.25	$C_{41}H_{59}N_7O_9$	3.98	X	X			X		Murakami et al. 1997
179	6	AP	AP D	Lys	Phe	$[M+H]^+$	828.4290	-0.12	$C_{44}H_{57}N_7O_9$	4.13	X				X	X	Mazur-Marzec et al. 2016
180	6	AP	FA A	Lys	Trp	$[M+H]^+$	867.4398	-0.11	$C_{46}H_{58}N_8O_9$	4.13						X	Williams et al. 1996
181	6	AP	AP 885	Lys	2-amino-5-hydroxy-phenylpentanoic acid	$[M+H]^+$	886.4708	-0.11	$C_{47}H_{63}N_7O_{10}$	4.22	X						McCann 2022
182	6	AP	FA B	Lys	Trp	$[M+H]^+$	881.4557	0.11	$C_{47}H_{60}N_8O_9$	4.43						X	Williams et al. 1996
183	6	AP	AP 807	Lys	Phe	$[M+H]^+$	808.4603	-0.12	$C_{42}H_{61}O_9N_7$	4.56	X						This study
184	6	AP	AP 841B	Lys	Phe	$[M+H]^+$	842.4449	0.24	$C_{45}H_{59}O_9N_7$	4.60	X						This study
185	6	AP	AP 856	Lys	2-amino-5-phenylpentanoic acid	$[M+H]^+$	856.4604	0.12	$C_{46}H_{61}O_9N_7$	5.03	X						Isaacs 2011
186	Ind. Node	AP	AP 867	Lys	Phe	$[M+H]^+$	868.3694	-0.23	$C_{37}H_{49}O_{12}N_{13}$	5.14			X	X			This study

187	6	AP	AP 850	Lys	Trp	$[M+H]^{+}$	851.4445	-0.59	$C_{46}H_{58}N_{8}O_{8}$	5.17						X	McDonald 2023
188	6	AP	AP 869	Lys	Phe	$[M+H]^{+}$	870.4758	-0.23	$C_{47}H_{63}O_{9}N_{7}$	5.25	X						This study
189	6	AP	AP 825B	Lys	Phe	$[M+H]^{+}$	826.4168	0.24	$C_{33}H_{55}O_{12}N_{13}$	5.45	X						This study
190	N/A	AP	AP 839	Lys	Phe	$[M+H]^{+}$	840.4652	-0.24	$C_{46}H_{61}O_{8}N_{7}$	5.59	X						This study
191	10	MV	MV 1653	Trp	Tyr	$[M+2H]^{2+}$	827.8459	0.24	$C_{81}H_{94}O_{21}N_{18}$	2.56		X				X	This study
192	10	MV	MV 1637	Trp	Tyr	$[M+2H]^{2+}$	819.8481	-0.12	$C_{81}H_{93}O_{20}N_{18}$	2.64		X				X	This study
193	10	MV	MV 1685	Trp	Tyr	$[M+2H]^{2+}$	843.8583	-0.24	$C_{95}H_{89}O_{11}N_{20}$	2.67		X				X	This study
194	10	MV	MV 1871	Trp	Tyr	$[M+2H]^{2+}$	936.8992	-0.11	$C_{80}H_{117}O_{34}N_{18}$	2.75		X					This study
195	N/A	MV	MV 1619	Trp	Tyr	$[M+2H]^{2+}$	810.8415	-0.12	$C_{77}H_{87}O_{17}N_{24}$	2.77		X					This study
196	10	MV	MV 1901	Trp	Tyr	$[M+2H]^{2+}$	951.9023	-0.11	$C_{77}H_{113}O_{33}N_{24}$	2.78		X				X	This study
197	10	MV	MV 1699	Trp	Tyr	$[M+2H]^{2+}$	850.8668	-0.12	$C_{70}H_{107}O_{33}N_{16}$	2.81	X					X	This study
198	10	MV	MV 1853	Trp	Tyr	$[M+2H]^{2+}$	927.8921	-0.10	$C_{105}H_{101}O_{15}N_{18}$	2.83		X					This study
199	10	MV	MV 1669	Trp	Tyr	$[M+2H]^{2+}$	835.8614	0.12	$C_{82}H_{97}O_{21}N_{18}$	2.84		X				X	This study
200	10	MV	MV 1933	Trp	Tyr	$[M+2H]^{2+}$	967.9169	0.10	$C_{95}H_{113}O_{25}N_{20}$	2.87						X	This study
201	10	MV	MV 1903	Trp	Tyr	$[M+2H]^{2+}$	952.9110	0.42	$C_{94}H_{111}O_{24}N_{20}$	2.88		X				X	This study
202	9	MV	MV 1521	Trp	Tyr	$[M+2H]^{2+}$	761.8185	-0.53	$C_{77}H_{85}O_{18}N_{16}$	2.89		X					This study
203	7	MV	MV 1529	Trp	Phe	$[M+2H]^{2+}$	765.8366	-0.52	$C_{74}H_{93}O_{22}N_{14}$	2.92	X				X		This study
204	10	MV	MV 1885	Trp	Phe	$[M+2H]^{2+}$	943.9064	0.21	$C_{94}H_{109}O_{23}N_{20}$	2.93		X					This study
205	N/A	MV	MV 1754	Trp	Tyr	$[M+2H]^{2+}$	878.3882	-0.23	$C_{87}H_{100}O_{18}N_{23}$	2.94		X				X	This study
206	10	MV	MV 1683	Trp	Tyr	$[M+2H]^{2+}$	842.8682	0.24	$C_{76}H_{104}N_{18}O_{26}$	2.96		X				X	This study
207	9	MV	MV 1553	Trp	Tyr	$[M+2H]^{2+}$	777.8319	-0.13	$C_{78}H_{89}O_{19}N_{16}$	2.97		X				X	This study
208	Ind. Node	MV	MV 1526	Trp	Tyr	$[M+2H]^{2+}$	764.3364	-0.13	$C_{71}H_{84}O_{15}N_{25}$	3	X				X		This study
209	9	MV	MV 1523	Trp	Tyr	$[M+2H]^{2+}$	762.8270	-0.07	$C_{77}H_{89}O_{18}N_{16}$	3.01						X	This study
210	N/A	MV	MV 1544	Trp	Tyr	$[M+2H]^{2+}$	773.3392	-0.39	$C_{81}H_{90}O_{17}N_{15}$	3.08					X		This study
211	N/A	MV	MV 1749	Trp	Lys	$[M+2H]^{2+}$	875.8772	-0.23	$C_{80}H_{105}O_{27}N_{18}$	3.22					X		This study
212	9	MV	MV 1505	Trp	Tyr	$[M+2H]^{2+}$	753.8214	-0.13	$C_{77}H_{85}O_{17}N_{16}$	3.23		X					This study
213	9	MV	MV 1537	Trp	Tyr	$[M+2H]^{2+}$	769.8345	-0.13	$C_{78}H_{89}O_{18}N_{16}$	3.28		X				X	This study
214	N/A	MV	MV 1494	Trp	Lys	$[M+2H]^{2+}$	748.3354	-0.27	$C_{72}H_{86}O_{14}N_{23}$	3.3		X					This study
215	7	MV	MV 1731	Trp	Phe	$[M+2H]^{2+}$	866.8727	-0.11	$C_{81}H_{99}O_{22}N_{22}$	3.37	X				X		This study
216	Ind. Node	MV	MV 1733	Trp	Phe	$[M+2H]^{2+}$	867.8798	-0.12	$C_{80}H_{105}O_{26}N_{18}$	3.53	X				X		This study
217	8	MV	MV 1747	Trp	Tyr	$[M+2H]^{2+}$	874.8876	-0.11	$C_{81}H_{107}O_{26}N_{18}$	3.75	X				X		This study

218	N/A	MV	MV 1753	Trp	Lys	$[M+2H]^{2+}$	877.8491	0.11	$C_{88}H_{89}O_{17}N_{24}$	3.75	X				X		This study
219	N/A	MV	MV 1730	Trp	Lys	$[M+2H]^{2+}$	866.3926	-0.12	$C_{82}H_{108}O_{25}N_{17}$	3.76						X	This study
220	8	MV	MV 1765	Trp	Lys	$[M+2H]^{2+}$	883.8928	-0.23	$C_{81}H_{111}O_{27}N_{18}$	3.78	X				X		This study
221	N/A	MV	MV 1737	Trp	Phe	$[M+2H]^{2+}$	869.8654	-0.11	$C_{78}H_{97}O_{23}N_{24}$	3.81	X				X		This study
222	7	MV	MV 1715	Trp	Phe	$[M+2H]^{2+}$	858.8754	0.12	$C_{81}H_{99}O_{21}N_{22}$	3.97	X				X		This study
223	8	MV	MV 1779	Trp	Lys	$[M+2H]^{2+}$	890.9010	0.11	$C_{82}H_{111}O_{27}N_{18}$	3.95	X				X		This study
224	N/A	MV	MV 1895	Trp	Tyr	$[M+2H]^{2+}$	948.9013	-0.11	$C_{88}H_{113}O_{28}N_{20}$	3.97					X		This study
225	N/A	MV	MV 1785	Trp	Lys	$[M+2H]^{2+}$	893.8629	-0.11	$C_{92}H_{101}O_{24}N_{14}$	4.11					X		This study

Abbreviations: AG; aeruginosin, AP; anabaenopeptin, CP; cyanopeptolin, FA; ferintoic acid, Lxx; leucine or isoleucine, MC; microcystin, MV; microviridin. Ind. Node indicates the compound appeared as an individual node within the GNPS molecular network. N/A indicates the compound did not appear within the molecular network.

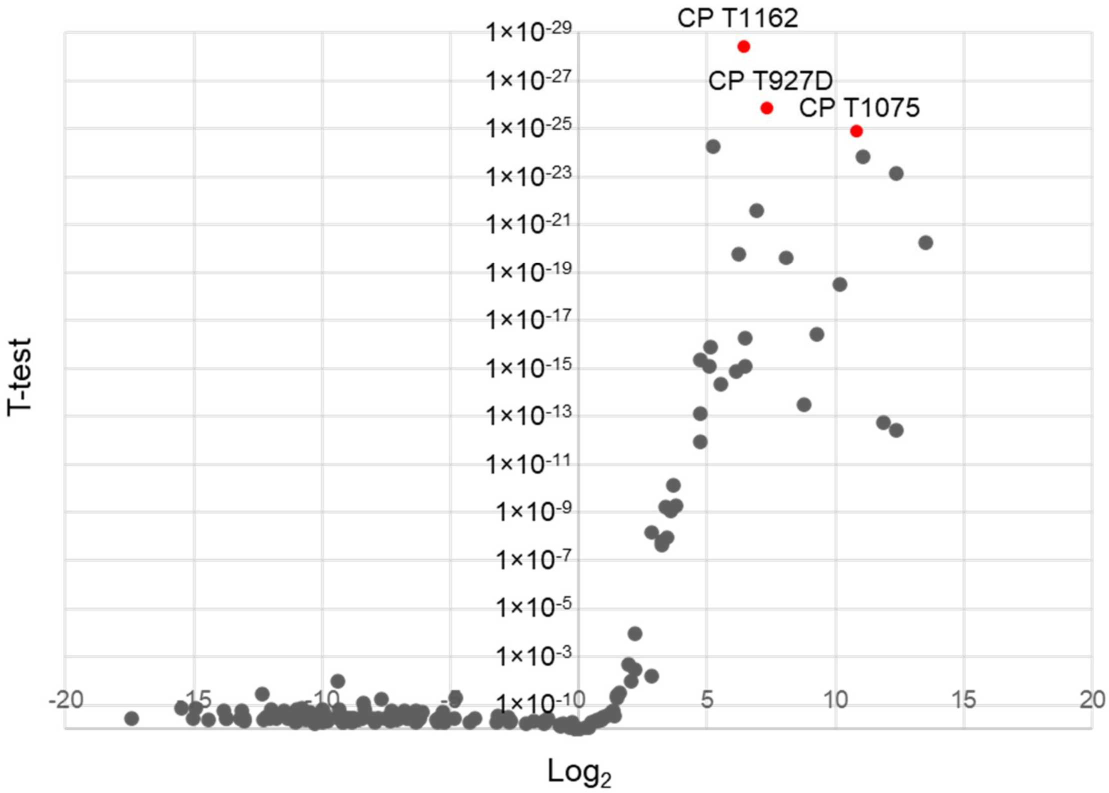

Figure S3. Volcano plot for *P. rubescens* CPCC 507 features.

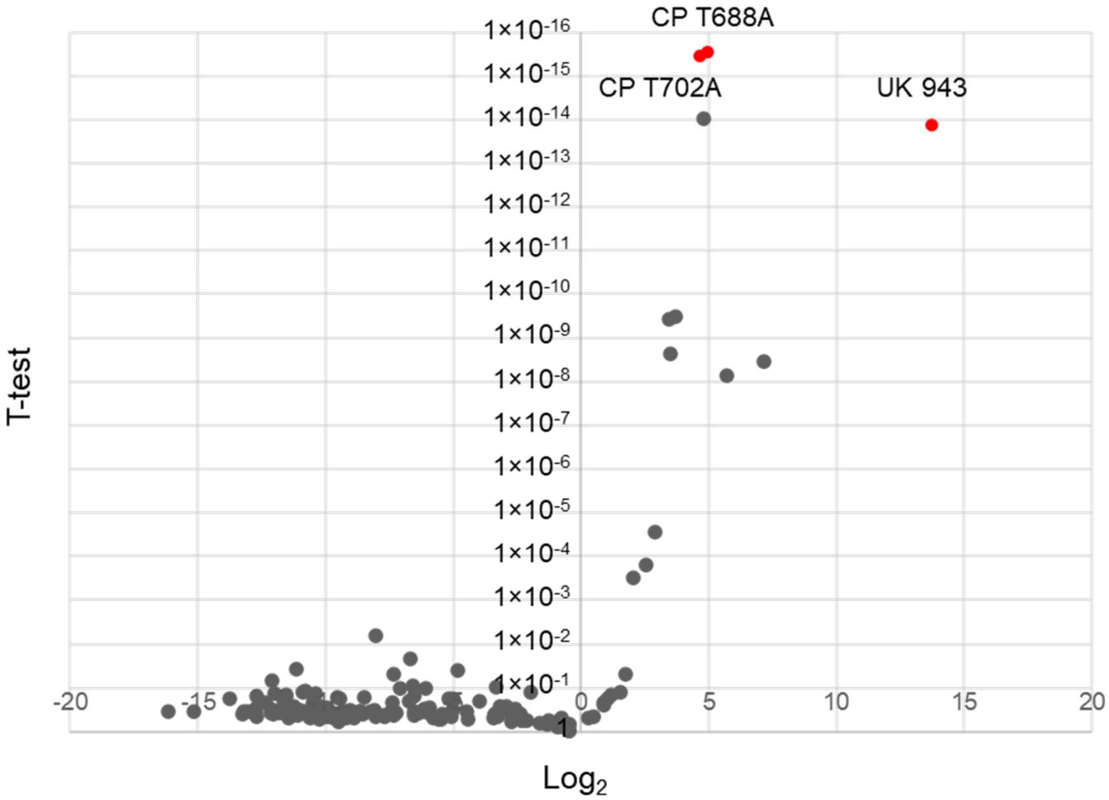

Figure S4. Volcano plot for *P. agardhii* CPCC 720 features.

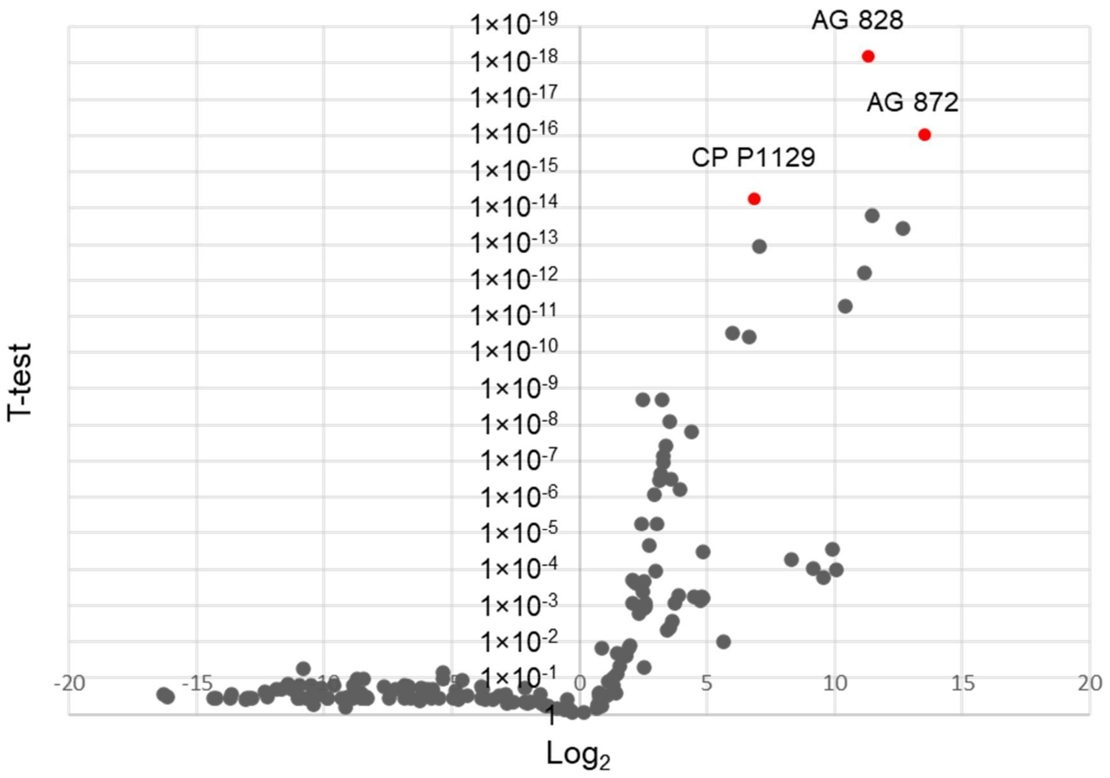

Figure S5. Volcano plot for *P. rubescens* CPCC 731 features.

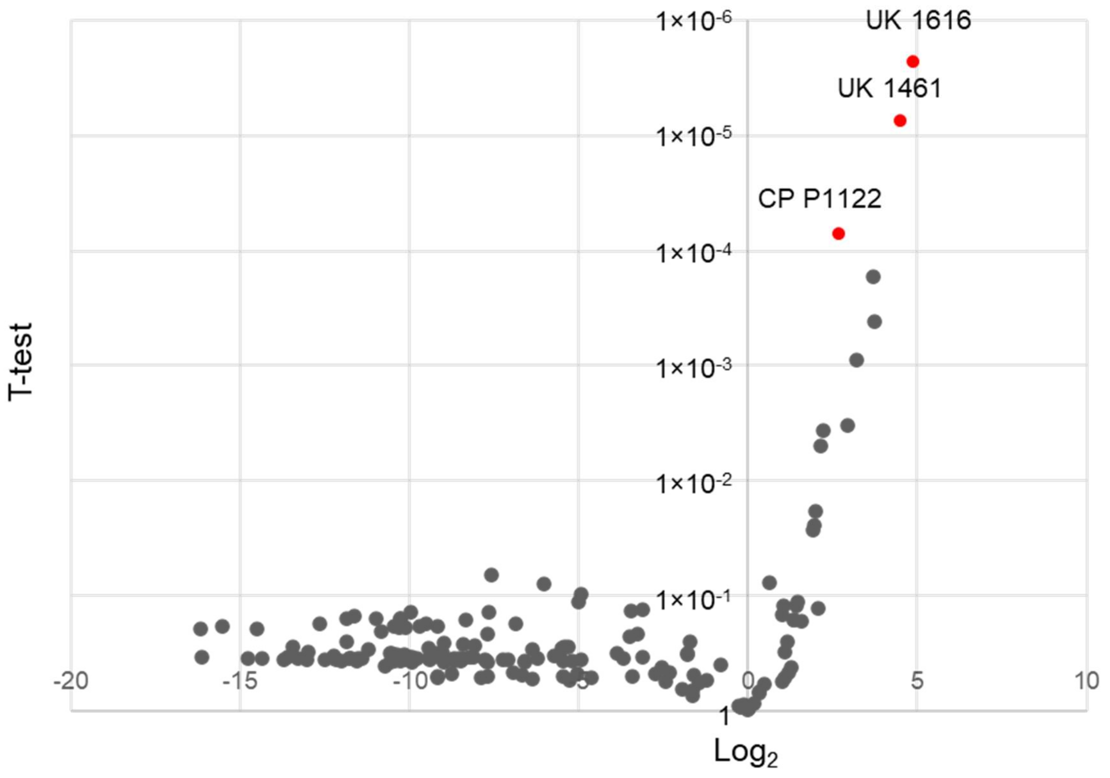

Figure S6. Volcano plot for *P. rubescens* CPCC 732 features.

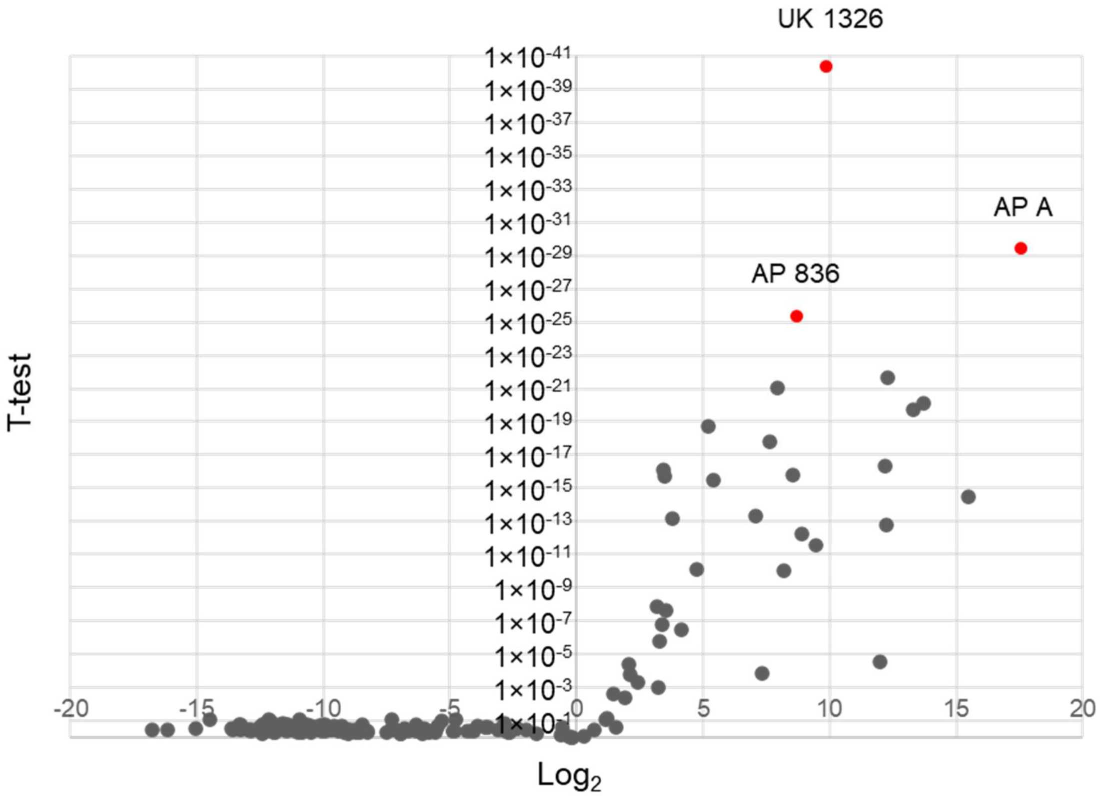

Figure S7. Volcano plot for *P. rubescens* CPCC 733 features.

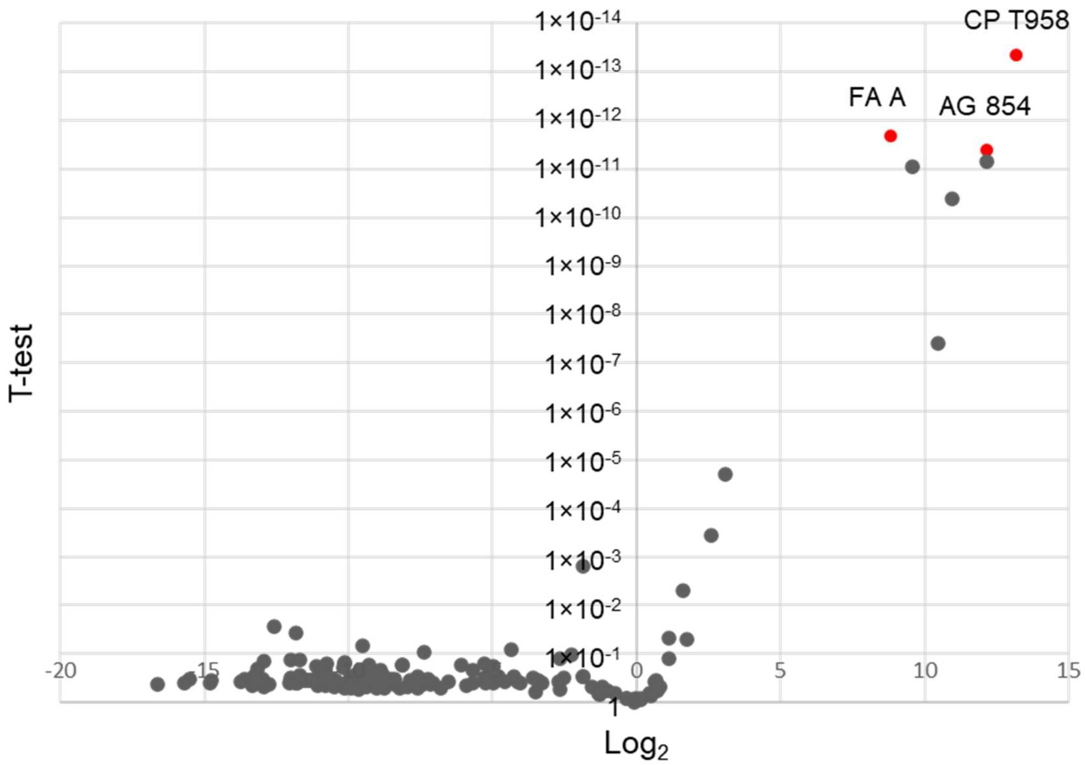

Figure S8. Volcano plot for *Planktothrix* sp. CPCC 735 features.

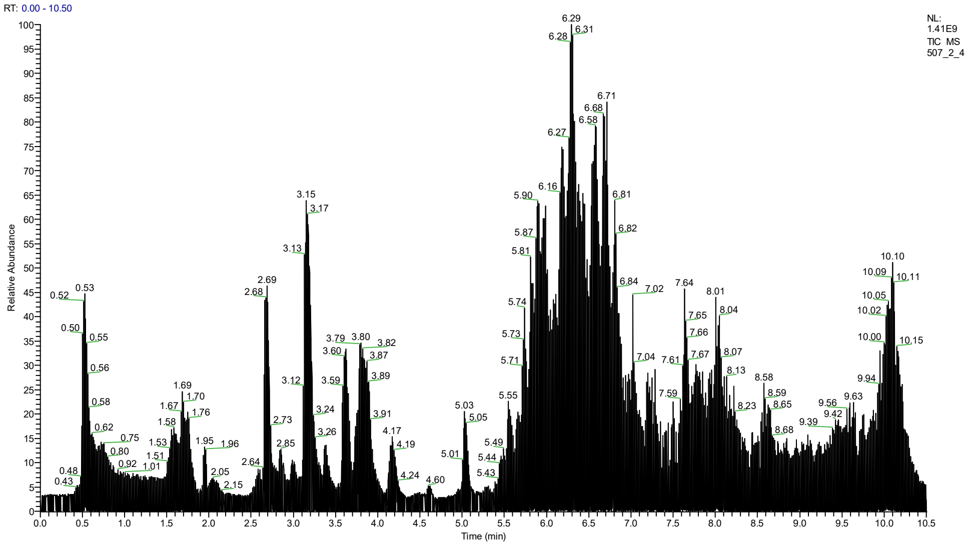

Figure S9. TIC for *P. rubescens* CPCC 507.

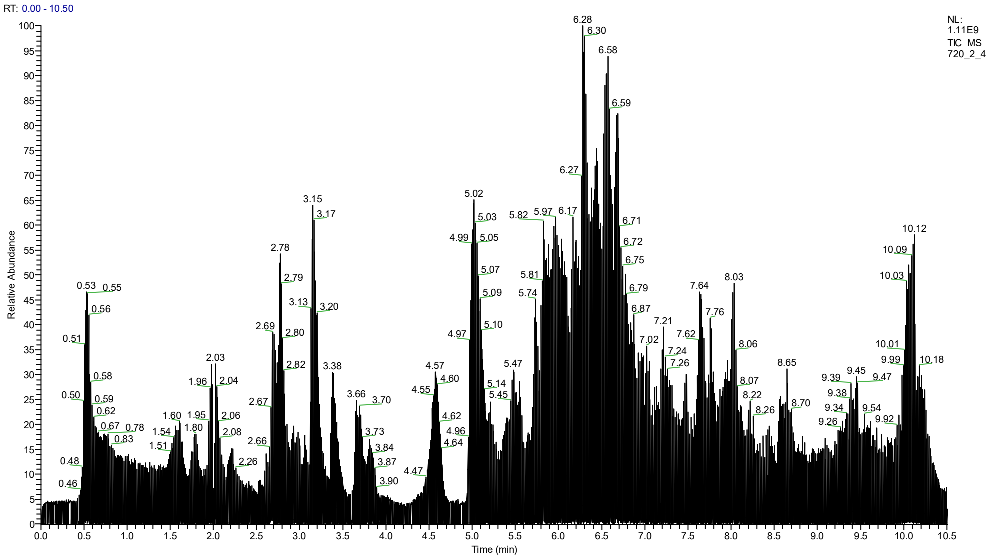

Figure S10. TIC for *P. agardhii* CPCC 720.

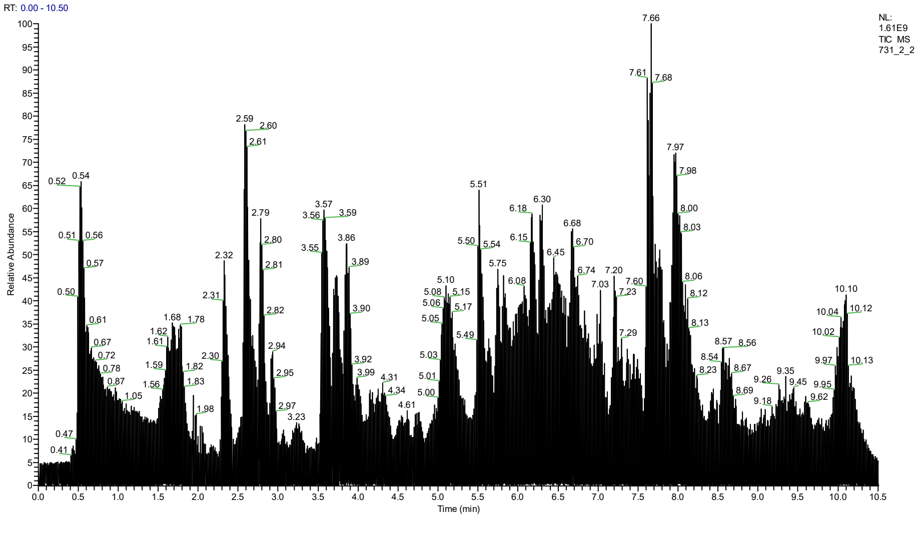

Figure S11. TIC for *P. rubescens* CPCC 731.

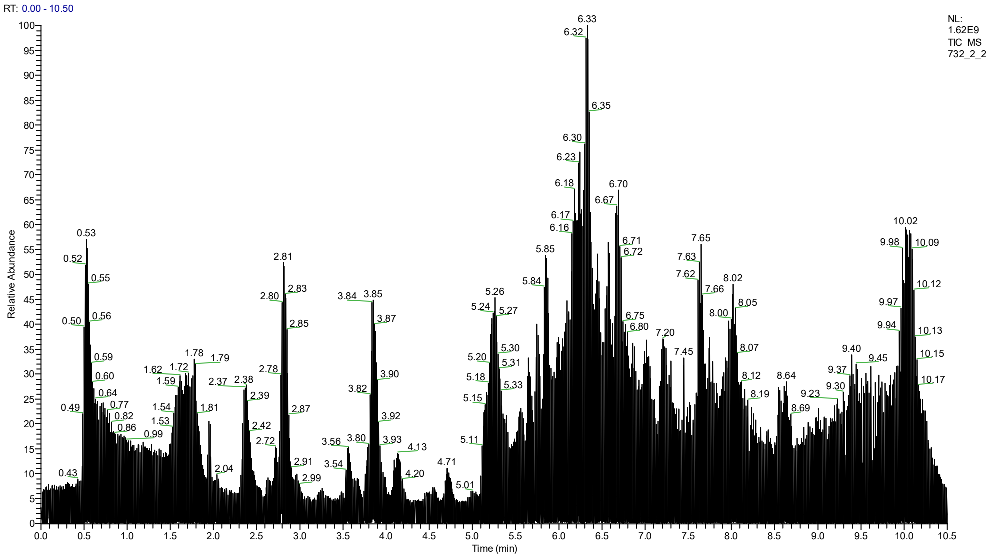

Figure S12. TIC for *P. rubescens* CPCC 732.

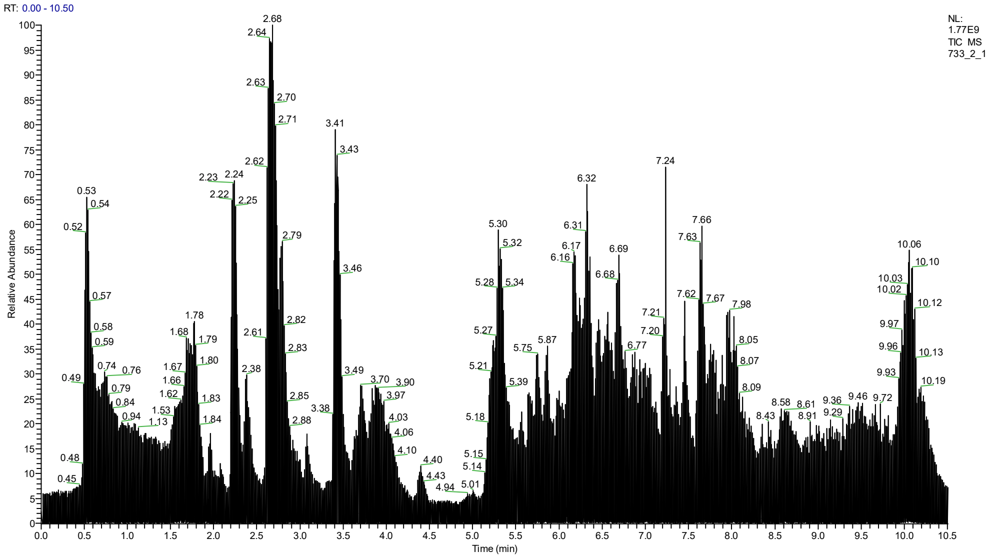

Figure S13. TIC for *P. rubescens* CPCC 733.

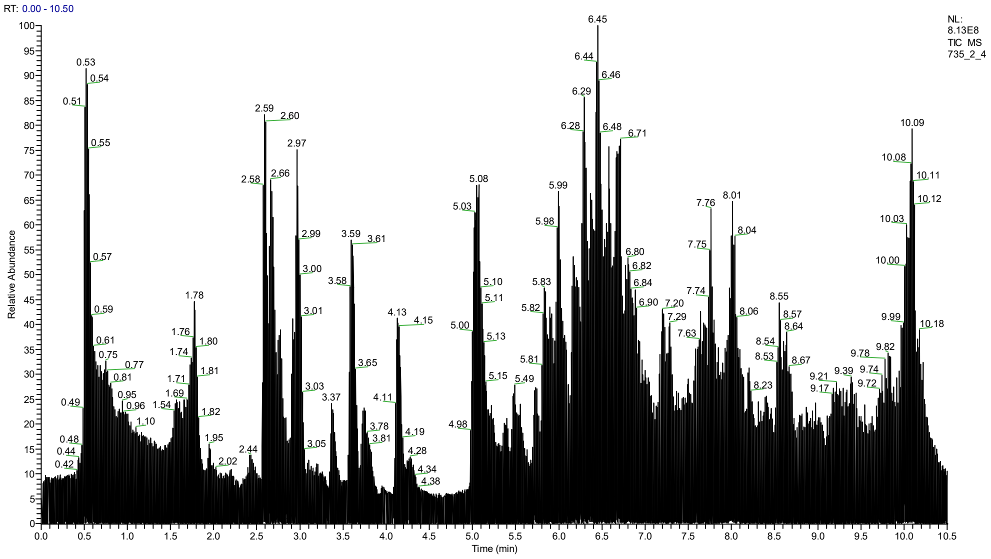

Figure S14. TIC for *Planktothrix* sp. CPCC 735.
